# One‐Pot Direct Recycling of Spent LiCoO_2_ via Synergistic Binder Defluorination and Phase Reconstruction

**DOI:** 10.1002/advs.76961

**Published:** 2026-08-03

**Authors:** Yingjie Hu, Songhu Ye, Fan Li, Chunhui Zhong, Bailin Xiang, Yulan Chen, Lili Zhi, Qingxia Liu, Junfeng Li, Zhixiang Chen

**Affiliations:** ^1^ Future Technology School Shenzhen Technology University Shenzhen China; ^2^ College of Physics and Materials Science Changji University Changji China; ^3^ College of Mining Engineering Taiyuan University of Technology Taiyuan China; ^4^ School of Chemical Engineering and Technology China University of Mining and Technology Xuzhou China

**Keywords:** deep eutectic solvent, direct recycling, spent lithium‐ion batteries, targeted defluorination

## Abstract

Direct recycling of spent lithium‐ion battery cathodes is hindered by two interdependent challenges: persistent polyvinylidene fluoride (PVDF) binder residues that block particle surfaces and incomplete structural repair of degraded layered phases. Here, we report a one‐pot regeneration strategy based on a Li^+^‐containing deep eutectic solvent (Li^+^‐DES) that integrates targeted defluorination and phase reconstruction within a reusable medium. Under mild conditions, the functionalized Li^+^‐DES selectively cleaves C─F bonds of PVDF, enabling complete binder removal and interface purification. Without any solvent exchange, the same Li^+^‐DES serves as a lithium‐rich repair medium, in which enhanced Li^+^ coordination and transport promote the formation of a uniform pre‐lithiation layer and facilitate the conversion of inactive spinel Co_3_O_4_ back into well‐ordered layered LiCoO_2_ upon annealing. The regenerated LiCoO_2_ delivers a discharge capacity of 157.4 mAh g^−1^ at 0.1 C, retains 83.1% of its capacity after 300 cycles, and maintains stable cycling even at 4.6 V. By integrating defluorination, Li replenishment, and structural reconstruction into a single reusable system, this work provides a simplified, energy‐efficient, and sustainable route for closed‐loop recycling of degraded cathode materials.

## Graphical abstract

A Li^+^‐DES system enables targeted defluorination and synergistic repair of spent LiCoO_2_ cathodes in a single medium. The regenerated material recovers a well‐ordered layered structure with a high capacity of 157.4 mAh g^−1^ and 83.1% capacity retention after 300 cycles, demonstrating a sustainable and efficient closed‐loop recycling strategy for lithium‐ion batteries.

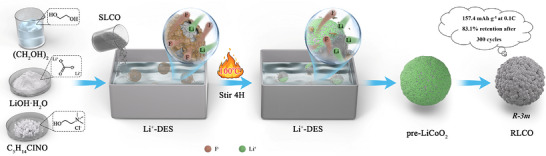

## 1 Introduction

The rapidly growing number of spent lithium‐ion batteries has created an urgent need for efficient, low‐carbon, and high‐value recycling technologies [1, 2, 3]. Direct regeneration, which repairs the degraded cathode material while preserving its original crystal framework, is considered a promising closed‐loop solution. However, its practical implementation faces two closely intertwined challenges [4, 5]. The highly stable polyvinylidene fluoride (PVDF) binder is difficult to remove under mild conditions without damaging the active materials [6]; and after prolonged cycling, lithium loss [7], lattice collapse [8, 9], and phase transformation [10] make it hard to achieve uniform and efficient structural recovery of the degraded cathodes. Conventional approaches tackle these issues in separate steps, a strategy that inevitably brings high energy consumption, heavy use of corrosive reagents, and lengthy processing.

Deep eutectic solvents (DESs) have attracted growing interest in battery recycling because of their low toxicity, tunable solvation properties, and ease of preparation [11]. Recent work has shown that DESs can leach metals or dissolve PVDF in a pretreatment stage, partially alleviating the environmental concerns of traditional acid–alkali systems [12, 13, 14, 15, 16, 17]. Nevertheless, previous DES‐based studies often lack a clear understanding of the impurity removal mechanism and fail to simultaneously achieve binder elimination, lithium replenishment, and structural repair in an integrated manner. Addressing this, the fundamental challenge lies in designing a DES‐based strategy that can realize all three functions synergistically.

Here, we overcome these limitations by developing a Li^+^‐DES that performs targeted defluorination and structural reconstruction in a single pot. The Li^+^‐DES selectively cleaves C─F bonds of PVDF under mild conditions, enabling complete binder removal and interface purification. Without any solvent exchange, the same system then acts as a Li‐rich repair medium, where the enhanced Li^+^ coordination and transport create a uniform pre‐lithiation layer on the cathode surface. The pre‐lithiated interface is key to the subsequent regeneration, it directs the conversion of inactive spinel Co_3_O_4_ into well‐ordered layered LiCoO_2_ during annealing while suppressing cation mixing and restoring lattice integrity. By integrating defluorination, Li replenishment, and structural reconstruction into a single reusable medium, this one‐pot strategy offers a streamlined, energy‐efficient, and sustainable route for closed‐loop recycling of spent lithium‐ion battery cathodes.

## 2 Results and Discussion

### 2.1 Targeted Defluorination in DES

**FIGURE 1:**
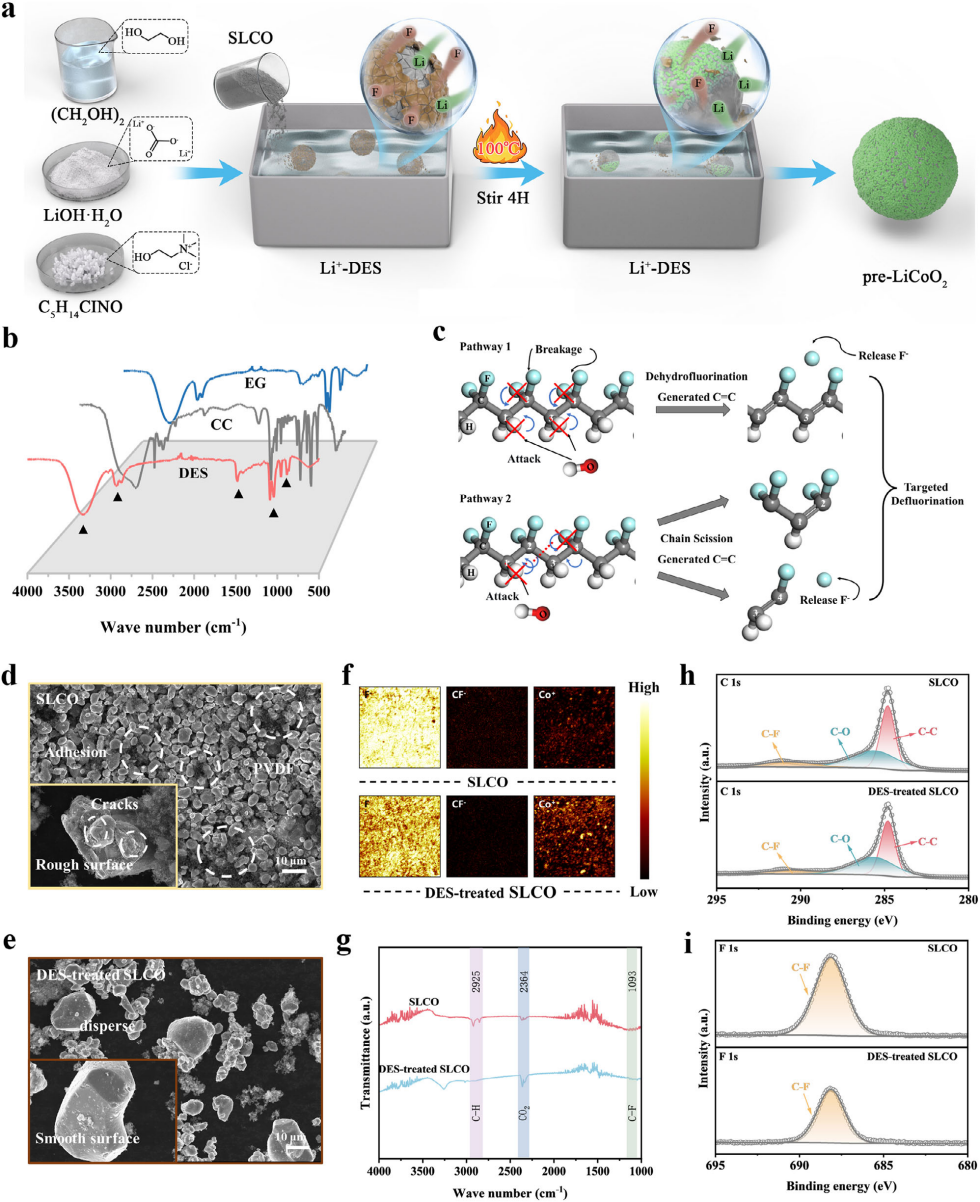
(a) Schematic Diagram of Li^+^‐DES Synthesis and Surface Reconstruction Process. (b) FTIR spectra of EG, CC, and DES. (c) Reaction Pathways for PVDF Degradation. SEM and magnified SEM images of (d) SLCO and (e) DES‐treated SLCO. (f) Comparison of Time‐of‐Flight Secondary Ion Mass Spectrometry (TOF‐SIMS) results for materials before and after DES treatment. (g) FT– IR spectra of SLCO and DES‐treated SLCO. The XPS patterns of SLCO and DES‐treated SLCO: (h) C 1s, (i) F 1s.

To overcome the resource waste and environmental pollution from conventional recycling methods, we propose a one‐pot direct recycling strategy using a Li^+^‐based deep eutectic solvent (Li^+^‐DES). Figure 1a illustrates the Li^+^‐DES treatment process for spent LiCoO_2_ (SLCO). The synthetic route of the DES is shown in Figure S1. The FT–IR spectrum of the DES (Figure 1b) reveals a broad, intense O─H band near 3300 cm^−1^ that is significantly shifted compared to the pure components, indicating strong intermolecular hydrogen bonding interactions in the DES network. Importantly, the characteristic ethylene glycol (EG) bands remain intact, confirming the formation of the DES without chemical decomposition of the components [18, 19]. Notably, the FT–IR spectrum of the recovered DES (Figure S2) is nearly superimposable onto that of the fresh DES. This spectral consistency, particularly in the O─H and C═O vibrational regions, indicates that the internal hydrogen‐bond network and multi‐component interactions are preserved upon recovery, demonstrating the structural stability and reusability of the DES [20, 21].

The Li^+^‐DES system assumes multiple active functions during the regeneration process, rather than serving merely as an inert solvent for Li^+^. In the liquid‐phase treatment stage, choline cations and ethylene glycol act synergistically to weaken the C─F bonds in PVDF, achieving selective and targeted defluorination, thereby removing the binder layer and purifying the particle surface. Meanwhile, the hydrogen‐bond network formed between Cl^−^ and ethylene glycol provides efficient transport pathways for Li^+^, enabling its rapid migration to the particle surfaces and preliminary intercalation into near‐surface vacancies [22]. Through this synergistic mechanism, the DES integrates the multifunctional roles of a defluorinating agent, an ion transport medium, and a pre‐lithiation mediator within a single system. The selection of (Choline chloride) CC and EG as the hydrogen bond acceptor/donor pair originates from their complementary electrostatic potentials. The high positive potential of Ch^+^ targets fluoride ions, while the σ‐hole of EG hydroxyls forms strong hydrogen bonds with chloride ions, synergistically enhancing the polarization capability [23]. Consequently, CC‐EG DES enables efficient defluorination under mild conditions without side reactions with the active components of SLCO, a balance rarely achieved with other HBA/HBD pairs.

The targeted defluorination mechanism mediated by Li^+^‐DES is illustrated in Figure 1c. In Pathway 1, OH^−^ from the DES extracts a β‐hydrogen from the PVDF main chain, generating a carbanion that undergoes β‐elimination. This induces heterolytic C─F bond cleavage, releasing F‐ and forming C═C bonds along the polymer backbone [23]. In Pathway 2, after initial F^−^ release, the weakened C─C bond undergoes preferential cleavage, and the resulting reactive chain‐end rapidly undergoes further β‐elimination, producing additional C═C bonds and releasing more F^−^. These two pathways act synergistically to achieve complete defluorination and structural disintegration of the PVDF binder [24].

Scanning electron microscopy (SEM) images (Figure 1d) show that the pristine SLCO particles are densely packed, a consequence of strong PVDF binding during electrode fabrication. After DES treatment, the particles exhibit clean and smooth surfaces (Figure 1e), indicating effective interfacial purification. EDS mapping (Figures S3and S4) confirms that the F content decreases from approximately 3.65 wt.% in SLCO to near‐zero after DES treatment. TOF‐SIMS analysis (Figure 1f) further shows a marked decrease in the signal intensities of F^−^ and CF^−^ fragments on the SLCO surface after DES treatment, accompanied by an increase in the Co^+^ signal.

FT–IR spectroscopy (Figure 1g) shows that the characteristic C─H and C─F peaks of PVDF at 2925 cm^−1^ almost disappear after Li^+^‐DES treatment. XPS analysis (Figure 1h,i and Figure S5) confirms a substantial decrease in the relative content of C─F bonds on the treated surface [25], while the proportion of lattice oxygen increases significantly. Collectively, these results provide unambiguous evidence for the efficient removal of the PVDF binder by the Li^+^‐DES system. To confirm that the defluorination results from direct C─F bond cleavage in PVDF rather than surface‐mediated reactions, pure PVDF powder was treated with the DES under identical conditions. As shown in Figure S6, FT–IR analysis reveals that the characteristic C─F absorption bands are significantly attenuated after DES treatment. These results confirm that the DES directly cleaves the C─F bonds of PVDF, independent of the SLCO surface chemistry.

Furthermore, when the recycled DES was applied to treat SLCO under the same conditions, XPS and SEM‐EDS analyses (Figures S7 and S8) demonstrated that the recycled DES retained a pronounced defluorination capability, confirming its reusability. Although the gradual accumulation of fluoride ions during extended cycling may affect the physicochemical properties of the DES, no significant deterioration in either structure or function was observed within the cycling regime examined in this work. For longer‐term reuse, periodic regeneration of the DES is recommended to sustain optimal performance.

### 2.2 Repair Mechanism of Degraded LCO

The regeneration process involves three stages (Figure S9): (I) solution‐phase pre‐lithiation, in which the Li^+^‐DES enables selective targeted defluorination and preliminary Li^+^ intercalation into the near‐surface region of the SLCO particles, accompanied by the healing of surface cracks; (II) annealing process, promoting Li^+^ diffusion from the near‐surface region of pre‐lithiated particles into the bulk lattice; and (III) structural restoration, in which the lithium‐deficient Li_1‐x_CoO_2_ is fully converted to stoichiometric LiCoO_2_ upon holding at 850°C, completing the reconstruction of the *R‐3m* layered structure.

**FIGURE 2:**
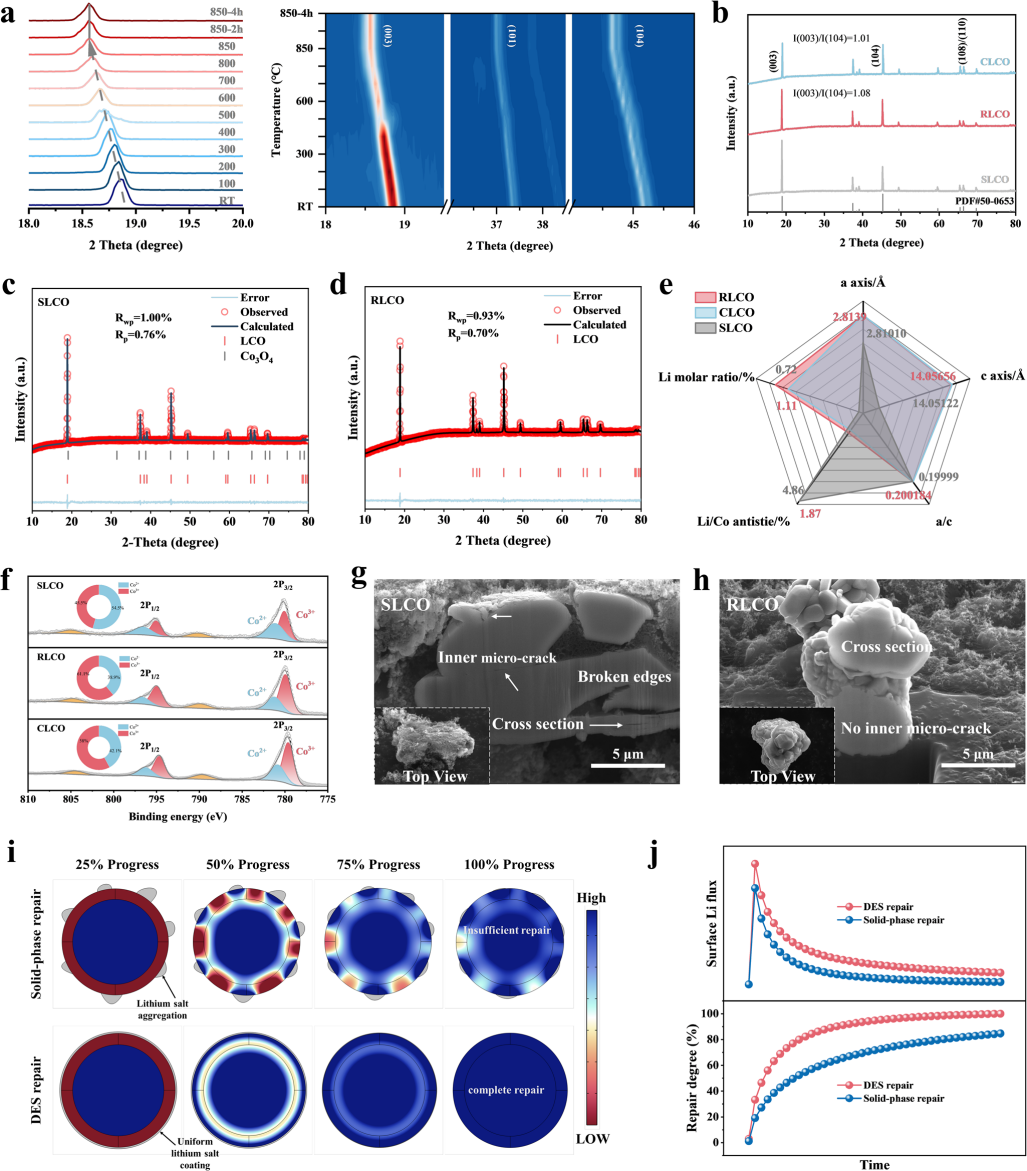
(a) In situ XRD patterns during annealing (RT: Room temperature). (b) XRD patterns of SLCO, RLCO, and CLCO. Rietveld refinement of the XRD pattern of (c) SLCO and (d) RLCO. (e) Comparison of key parameters of SLCO, RLCO, and CLCO. (f) XPS spectra of SLCO, RLCO, and CLCO. FIB‐SEM images of (g) SLCO and (h) RLCO. (i) Li^+^ replenishment and repair process through COMSOL simulations. (j) Graph of lithium flux and repair degree.

After defluorination, the same Li^+^‐DES environment serves as a Li‐rich medium. Under mild heating (100°C), the Li^+^‐DES promotes rapid Li^+^ transport to the cathode particle surfaces, forming a uniform pre‐lithiation layer. This pre‐lithiated surface is crucial for the subsequent annealing‐driven structural restoration. Figure 2a presents in situ XRD monitoring of the phase evolution during annealing. As the temperature increased from 600°C to 850°C, the (003), (101), and (104) diffraction peaks systematically shifted toward lower angles, indicating successful pre‐lithiation of the material [26]. After 4 h at 850°C, the (003) peak position stabilizes, marking the completion of the phase reconstruction. The regenerated LiCoO_2_ (RLCO) exhibits an I(003)/I(104) intensity ratio of 1.08, comparable to that of commercial LCO (CLCO, 1.01), confirming the recovery of a well‐ordered layered structure (Figure 2b) [27]. Rietveld refinement (Figure 2c–e, Figure S10 and Tables S1–S3) shows that RLCO has lattice parameters (a and c) closely matching those of CLCO, and the Li/Co cation mixing ratio is significantly reduced from 4.86% to 1.87%. These structural parameters collectively verify the effectiveness of the Li^+^‐DES‐assisted regeneration process.

ICP‐OES analysis (Figure S11) reveals that the Li/Co molar ratio increases from 0.72 (SLCO) to 1.11 (RLCO), closely approaching the reference value of 1.05 for commercial CLCO. XPS analysis of the RLCO surface detects only a trace amount of LiF (Figure S12), confirming that the majority of fluoride ions were stabilized in the DES matrix rather than depositing on the particles. This side reaction does not compromise the restored stoichiometry, because the lithium source was supplied in sufficient excess to compensate for the negligible lithium loss. The structural restoration of RLCO and its electrochemical performance further confirms that this trace LiF has no measurable impact on the regenerated material. XPS of the Co 2p (Figure 2f) shows that the relative content of Co^3+^ increased from 45.46% (SLCO) to 61.07% (RLCO), comparable to the 57.95% observed in CLCO. SEM and FIB‐SEM imaging (Figure 2g,h and Figure S13) visually confirm the morphological restoration, the degraded SLCO particles exhibited internal cracks and layered delamination, while the RLCO particles showed smooth surfaces and intact cross‐sections.

Furthermore, COMSOL simulations were employed to investigate the influence of two Li^+^ transport modes on the Li^+^ concentration gradient near the cathode material (Figure 2i). In the solid phase repair system, uneven grinding of the lithium salt leads to localized lithium‐source aggregation and deficiency on the cathode particle surface during pre‐lithiation, compromising the subsequent annealing step. Consequently, despite the overall abundance of lithium, complete repair of the cathode material is not achieved. In contrast, in the Li^+^‐DES system, thermally driven uniform coating of the lithium source onto the cathode particle surfaces enables rapid Li^+^ replenishment during pre‐lithiation, forming a pre‐lithiation layer that provides ample lithium for the subsequent annealing process, thereby enhancing surface lithium flux and repair efficiency (Figure 2j).

**FIGURE 3:**
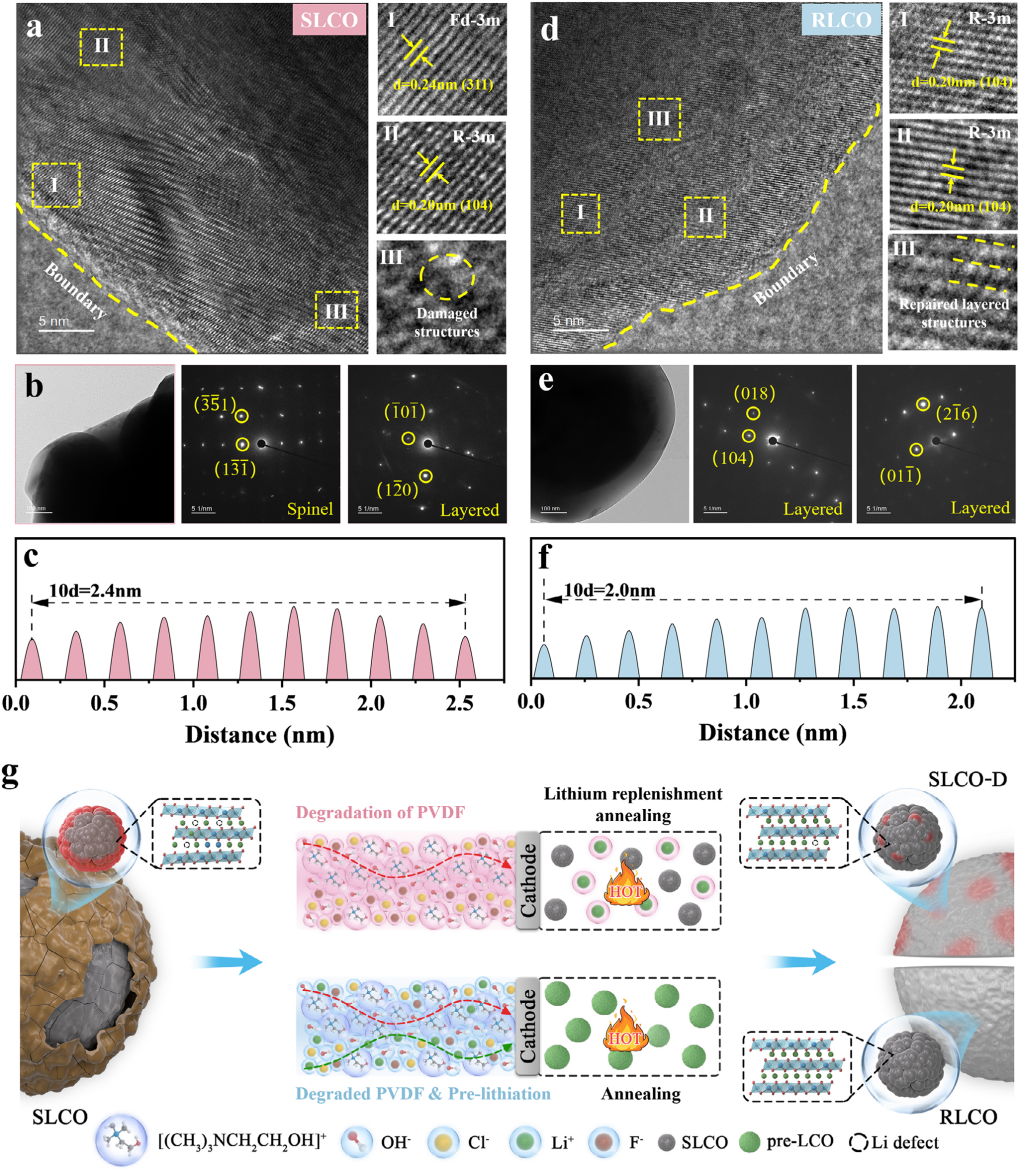
HRTEM analysis of (a–c) SLCO and (d–f) RLCO. Area (I, II, III) represents the FFT images of SLCO and RLCO. For SLCO, the bulk of the degraded particles remains a layered structure, but a spinel phase was observed in the bulk, while for RLCO, the bulk and surface structures of the repaired particles are consistent with a layered phase. Scale bars, 5 nm. SAED images of (b) SLCO and (e) RLCO. (g) Schematic of the repair mechanism.

To further elucidate the microstructural evolution during the cathode regeneration process, high‐resolution transmission electron microscopy (HRTEM) was employed to analyze three representative regions of the material. HRTEM analysis (Figure 3a) reveals the structural heterogeneity in SLCO. In the near‐surface region (Region I), lattice fringes of approximately 0.24 nm (Figure 3c) correspond to the (311) plane of spinel Co_3_O_4_, while the bulk interior (Region II) retains the layered LiCoO_2_ structure (0.20 nm) [28]. Meanwhile, Region III displays extensive structural disorder, attributed to lattice collapse resulting from repeated Li^+^ insertion/extraction during prolonged cycling. Correspondingly, the selected area electron diffraction (SAED) pattern reveals the presence of spinel Co_3_O_4_ within the SLCO matrix (Figure 3b).

Conversely, the HRTEM image of the RLCO displays distinct and periodic lattice fringes (Figure 3d). Detailed analysis of three selected regions (Regions I–III) confirms the successful restoration of the layered structure. The measured fringe spacing of approximately 0.20 nm (Figure 3f) aligns precisely with the (104) plane of layered LiCoO_2_, corroborated by the corresponding SAED pattern (Figure 3e). However, in contrast to the liquid‐phase regenerated sample, the HRTEM image of the solid‐state repaired sample (SLCO‐D, Figure S14) reveals that the layered ordered structure is only partially recovered.

Figure 3g illustrates the regeneration mechanism of the DES‐based repair system. In the Li^+^‐DES environment, the DES first removes surface PVDF, thereby purifying the material surface. Subsequently, under thermally assisted repair conditions, Li^+^ transport is accelerated, and ion transport kinetics are significantly enhanced, promoting the formation of a uniform pre‐lithiation layer. This sequential process ultimately leads to superior regeneration performance. In contrast, in the solid‐state repair system, the inhomogeneous pre‐lithiation layer results in limited structural recovery. Consequently, this one‐pot Li^+^‐DES strategy offers an efficient, low‐energy, and environmentally benign route for direct cathode regeneration, overcoming the limitations of solid‐state repair and enabling high‐performance material recovery.

### 2.3 Electrochemical Performance of Regenerated Cathode Materials

**FIGURE 4:**
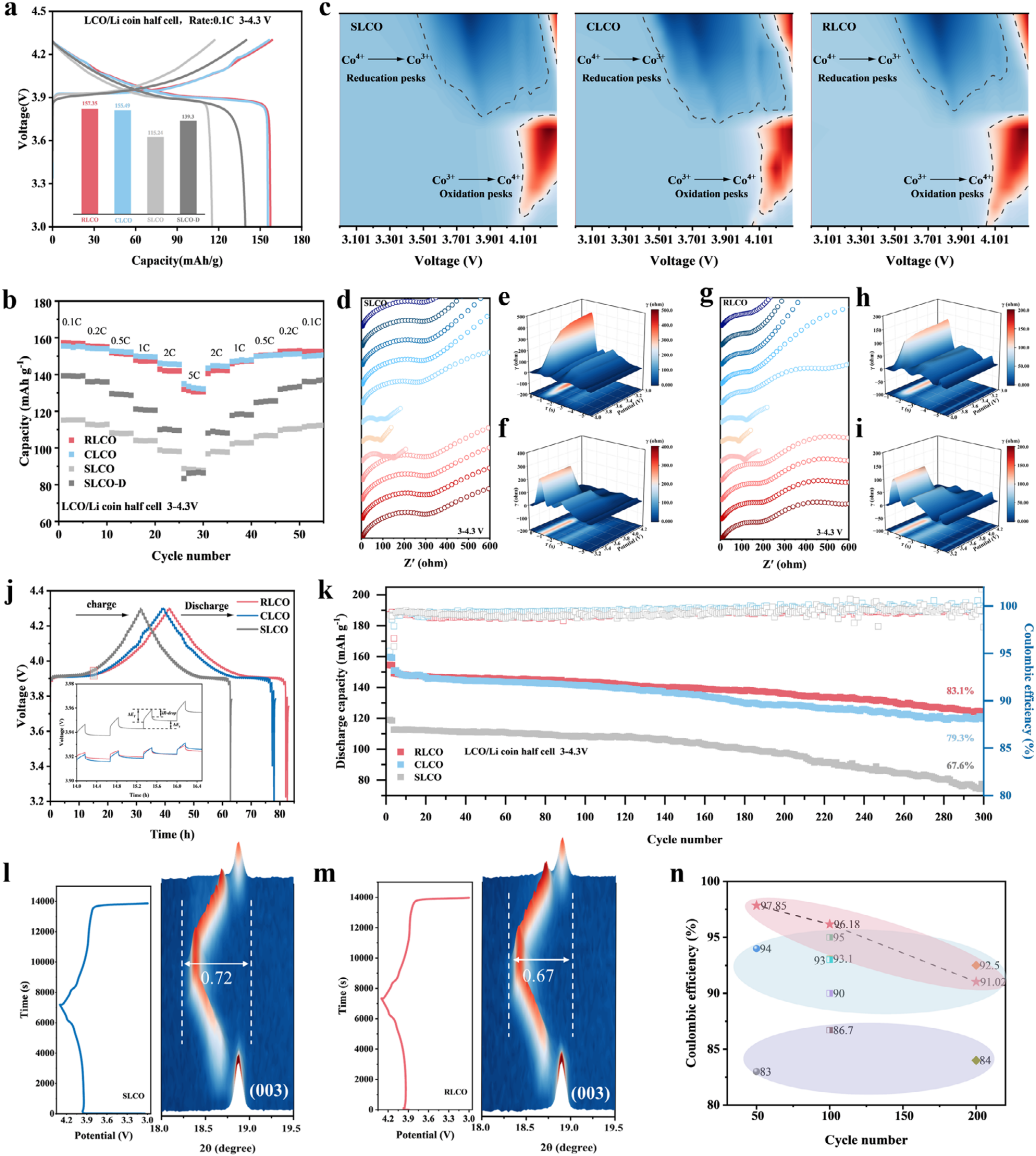
(a) Charge–discharge curves of SLCO, SLCO‐D, RLCO, and CLCO. (b) Rate performance of SLCO, SLCO‐D, RLCO, and CLCO. (c) Contour plots of the CV curves for SLCO, CLCO, and RLCO at different scan rates (0.1–1 mV s^−1^). In situ EIS Nyquist plots and the corresponding DRT results for (d–f) SLCO and (g–i) RLCO. (j) GITT curves of SLCO, CLCO, and RLCO. (k) Cycling performance of SLCO, SLCO‐D, RLCO, and CLCO. In situ XRD measurements during the initial cycle for (l) SLCO and (m) RLCO. (n) Comparison of the cycling performance of RLCO with regenerated cathode materials from the literature.

The electrochemical performance was evaluated in the voltage window 3–4.3 V. As shown in Figure 4a, the SLCO delivered a limited discharge capacity of 115.2 mAh g^−1^. After regeneration, RLCO exhibits a markedly higher capacity (157.4 mAh g^−1^), even surpassing that of pristine CLCO (155.5 mAh g^−1^) due to the lower Li/Co cation mixing [29]. In contrast, the solid‐state repaired SLCO‐D shows inferior performance (139.4 mAh g^−1^), underscoring the critical role of the Li^+^‐DES‐enabled pre‐lithiation step. Concurrently, to establish the optimal conditions for this regeneration strategy, we systematically evaluated the influence of key parameters. Optimization studies (Figures S15–S17) identify 15 wt.% Li supplement, 850°C sintering temperature, and 4 h stirring as the optimal conditions.

This capacity improvement is further reflected in the rate capability and redox reversibility. Rate capability measurements (Figure 4b) show that RLCO delivers specific capacities of 151.9, 147.4, 142.8, and 130.6 mAh g^−1^ at 0.5C, 1, 2C, and 5C, respectively. When the current density is returned to 0.1C, the capacity recovers to 152.6 mAh g^−1^, indicating excellent structural reversibility. Cyclic voltammetry (CV) profiles (Figure 4c and Figure S18) show that RLCO displays well‐defined and symmetric redox peaks with narrower peak widths than SLCO and SLCO‐D, indicating lower potential polarization and enhanced electrochemical reversibility. In situ electrochemical impedance spectroscopy (EIS) coupled with distribution of relaxation times (DRT) [30, 31] analysis (Figure 4d–i) reveals that RLCO exhibits a lower and more stable interfacial resistance (100–200 Ω) compared to SLCO (100–400 Ω). DRT analysis further shows that RLCO maintains consistently smaller and more stable characteristic relaxation times (τ) and amplitudes (γ) during cycling, indicating that the Li^+^‐DES process facilitates the formation of a more uniform and stable cathode‐electrolyte interphase.

Galvanostatic intermittent titration technique (GITT) measurements (Figure 4j) show that the regenerated cathode delivers a higher discharge capacity than CLCO and SLCO. Moreover, the lower ratio of steady‐state voltage change to total voltage variation (ΔEs/ΔEt) for RLCO indicates diminished polarization during lithiation/ delithiation. Consistently, the Li^+^ diffusion coefficients derived from GITT profiles (Figure S19) closely approach those of CLCO, confirming its superior Li^+^ transport kinetics in the regenerated material. The superior kinetics and stable interface of RLCO directly translate to enhanced long‐term cycling stability, as shown in Figure 4k. SLCO retains only 67.6% of its capacity after 300 cycles, whereas RLCO maintains 83.1% capacity retention with an initial capacity increase of 42.1 mAh g^−1^.

The underlying mechanism for these electrochemical improvements lies in the successful restoration of the layered structure. In situ XRD was performed during the first cycle to probe phase evolution (Figure 4l,m). RLCO exhibits significantly lower lattice strain (0.67°) than SLCO (0.71°) during cycling. The well‐ordered layered framework of RLCO mitigates lattice distortion during the H1‐to‐H2 phase transition and alleviates stress accumulation, thereby suppressing structural degradation. Given its robust and stable layered structure, RLCO was further subjected to high‐voltage testing. Elevating the charge cutoff voltage to 4.6 V enables more Li^+^ extraction, thereby increasing energy density. Remarkably, RLCO maintains good cycling stability under this demanding condition, delivering a discharge capacity of 206.3 mAh g^−1^ at 0.1 C (Figure S20) and retaining 70.4% of its capacity after 100 cycles at 0.5 C (Figure S21). Compared with recently reported regenerated LiCoO_2_ cathodes, the material restored via the DES‐based strategy exhibits competitive electrochemical performance (Figure 4n and Table S4) [21, 32, 33, 34, 35, 36, 37, 38, 39]. Therefore, these results confirm that the Li^+^‐DES regeneration strategy not only enables targeted defluorination but also induces structural reconstruction, thereby restoring and enhancing the electrochemical performance of SLCO. This provides a scalable and sustainable route for the recycling of high‐performance cathode materials.

### 2.4 Techno‐Economic Analysis

**FIGURE 5:**
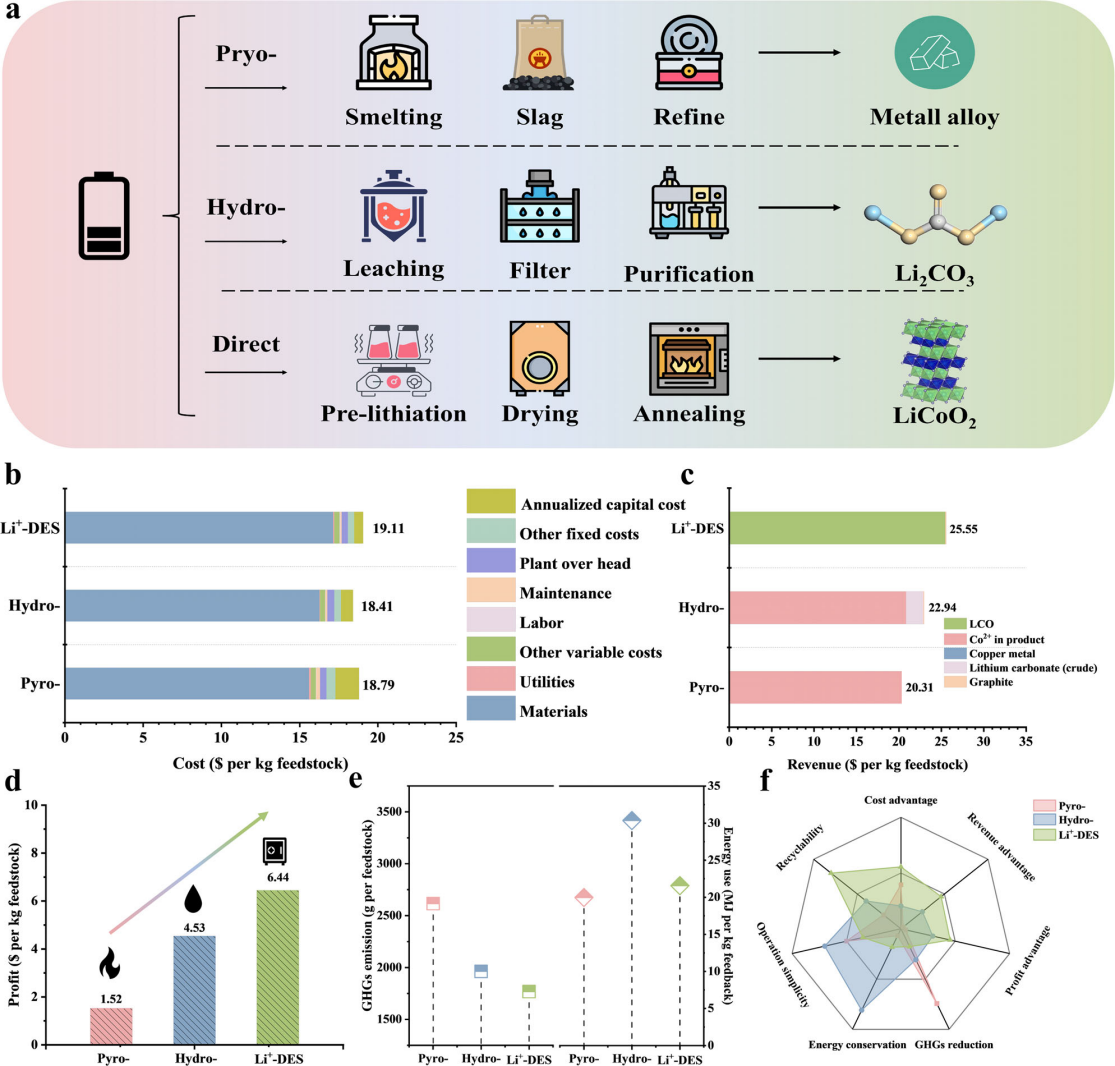
(a) Comparison of pyrometallurgical, hydrometallurgical, and Li^+^‐DES direct recycling processes for spent LCO cathodes. (b–f) Techno‐economic analysis results of the three recycling routes. (b) Cost analysis. (c) Revenue analysis. (d) Profit analysis. (e) Greenhouse gas (GHG) emissions and total energy consumption. (f) Comprehensive comparison of different battery recycling technologies.

Based on the Everbatt 2023 model, this study conducted a techno‐economic analysis (TEA) of different battery recycling technologies to evaluate the practical feasibility of the proposed Li^+^‐DES direct recycling route (Figure 5a and Tables S5–S11). The pyrometallurgical (Pyro‐) employs high‐temperature smelting to recover a Cu‐Co alloy, with a total cost of 18.79 USD·kg^−1^ and a net profit of 1.52 USD·kg^−1^. The hydrometallurgy (Hydro‐), which recovers Li_2_CO_3_ through acid leaching followed by multi‐step ion separation, achieves a slightly higher economic return of 4.53 USD·kg^−1^ but remains constrained by high acid consumption and operational complexity. In contrast, the Li^+^‐DES route produces RLCO as its main product, delivering the highest economic return of up to 6.44 USD·kg^−1^ (Figure 5b–d). Notably, this method avoids the use of strong acids and explosive reagents, thereby simplifying the operation and reducing hazardous waste generation. A life‐cycle environmental impact assessment further highlights the advantages of the Li^+^‐DES route, showing significantly lower greenhouse gas emissions and reduced energy consumption compared to both pyro‐ and hydro‐processes (Figure 5e). The three battery recycling technologies have been comprehensively compared, as summarized in Figure 5f. Collectively, these findings demonstrate that the proposed Li^+^‐DES direct recycling strategy offers superior economic returns and environmental sustainability, providing a promising alternative for the efficient and scalable recycling of spent cathode materials.

## 3 Conclusion

We have developed a one‐pot direct regeneration strategy based on a Li^+^‐DES that achieves synergistic binder defluorination and phase reconstruction of spent LiCoO_2_ cathodes. The key advance is the multifunctional integration of binder removal and structural repair within a single, reusable DES medium. Under mild conditions, the Li^+^‐DES selectively cleaves C─F bonds of PVDF, enabling complete binder removal and interface purification. Without solvent exchange, the same DES system then serves as a Li‐rich repair medium, promoting uniform pre‐lithiation and directing the conversion of inactive spinel Co_3_O_4_ into a well‐ordered layered LiCoO_2_ structure upon annealing. The regenerated cathode delivers a discharge capacity of 157.4 mAh g^−1^ at 0.1 C with 83.1% capacity retention after 300 cycles, and maintains stable cycling even at 4.6 V. Beyond LiCoO_2_, this DES‐based strategy is potentially extendable to other spent layered oxide cathodes (e.g., NCM and NCA) with a similar structure. By integrating defluorination, lithium replenishment, and structural reconstruction into a single reusable system, this work provides a simplified, energy‐efficient, and sustainable pathway for closed‐loop, high‐value recycling of lithium‐ion batteries.

## Author Contributions

**Yingjie Hu**: conceptualization, investigation, Writing – original draft, methodology, data curation. **Junfeng Li**: writing – original draft, conceptualization, investigation, data curation, supervision, methodology, writing – review and editing. **Zhixiang Chen**: funding acquisition, writing – original draft, writing – review and editing, data curation, supervision, methodology, investigation. **Yulan Chen**: methodology, data curation. **Bailin Xiang**: methodology, data curation. **Qingxia Liu**: supervision, writing – review and editing. **Songhu Ye**: investigation, methodology, data curation. **Fan Li**: methodology, conceptualization. **Chunhui Zhong**: investigation, methodology. **Lili Zhi**: supervision, writing – original draft, writing – review and editing, investigation.

## Conflicts of Interest

The authors declare no conflicts of interest.

## Supporting information




**Supporting File**: advs76961‐sup‐0001‐SuppMat.docx.

## Data Availability

The data that support the findings of this study are available from the corresponding author upon reasonable request.
