# Supplementary material for: One‐Pot Direct Recycling of Spent LiCoO2 via Synergistic Binder Defluorination and Phase Reconstruction
Source: Adv Sci (Weinh). 2026 Aug 3:e76961. Online ahead of print. doi: 10.1002/advs.76961 (PMC13430931; doi:10.1002/advs.76961)
Supplement: Supplementary file 1 — Supporting File: advs76961‐sup‐0001‐SuppMat.docx. [file ADVS-9999-e76961-s001.docx]

**Supporting Information**

**One-Pot Direct Recycling of Spent LiCoO_2_ via Synergistic Binder Defluorination and Phase Reconstruction**

Yingjie Hu ^a,b^, Songhu Ye ^a^, Fan Li ^a,c^, Chunhui Zhong ^a,d^, Bailin Xiang ^a^, Yulan Chen ^a^, Lili Zhi ^b*^, Qingxia Liu ^a^, Junfeng Li ^a*^, Zhixiang Chen ^a*^

*^a^ Future Technology School, Shenzhen Technology University, Shenzhen, 518055, China.*

*^b^ College of Physics and Materials Science, Changji University, Changji, 831100, China.*

*^c^ College of Mining Engineering, Taiyuan University of Technology, Taiyuan 030024, China*

*^d^ School of Chemical Engineering and Technology, China University of Mining and Technology, Xuzhou 221116, China*

# **Experimental Section**

**Purify Spent LiCoO_2_ (SLCO) Cathode Materials**

**DES-treated SLCO:** The targeted removal of the polyvinylidene fluoride (PVDF) binder was carried out using a tailor-made deep eutectic solvent (DES). The DES was synthesized by mixing ethylene glycol (EG, C_2_H_6_O_2_, ≥99%, Aladdin) and choline chloride (CC, C_5_H_14_ClNO, ≥98%, Aladdin) at a molar ratio of 1:2, followed by magnetic stirring at 60 °C for 1 h until a homogeneous, transparent liquid formed. Subsequently, the obtained SLCO black powder was introduced into the as-prepared DES and reacted under continuous stirring, achieving the efficient and selective dissolution of the PVDF binder.

**Regeneration of SLCO (SLCO-D and RLCO)**

**SLCO-D Preparation:** Recycled SLCO and lithium hydroxide monohydrate (LiOH·H_2_O, Aladdin) were mixed at a mass ratio of 1:0.15 and thoroughly homogenized by grinding in an agate mortar. The resulting precursor was then subjected to a solid-state sintering process under an air atmosphere. The sintering protocol included heating from room temperature to 850  °C at a ramp rate of 5  °C·min^-1^, holding for 4  h, and finally cooling naturally inside the furnace. The obtained product was designated as SLCO-D for further characterization.

**RLCO Preparation:** The obtained cathode black powder was directly added to a Li^+^-DES solution prepared by mixing LiOH·H_2_O and DES in a specific ratio. The mixture was continuously stirred and reacted at 100 °C for 4 hours. After the reaction, the mixture was filtered and washed three times with deionized water, then dried overnight in a forced-air drying oven. The pre-lithiated dried powder (rLCO) underwent high-temperature annealing in an air atmosphere: heated at a rate of 5 ℃/min from room temperature to 850 ℃, held at this temperature for 4 hours, and finally cooled naturally with the furnace to room temperature, yielding the final regenerated LCO material (RLCO).

**Coin Cell Assembly**

Electrochemical performance was evaluated using CR2032-type coin cells. The cathode slurry was prepared by mixing the active material (LCO), conductive carbon black (Super P), and polyvinylidene fluoride (PVDF) binder at a mass ratio of 8:1:1 in N‑methyl‑2‑pyrrolidone (NMP). The slurry was uniformly cast onto carbon-coated aluminum foil and dried at 110  °C in a vacuum oven for 12  h. Circular electrodes with a diameter of 12  mm were then punched from the dried sheet. The mass loading of the active material was controlled in the range of 2-3  mg cm^-2^. The coin cells were assembled in an argon‑filled glove box with moisture and oxygen levels maintained below 0.01  ppm. Each cell was configured with the prepared cathode, a lithium plate anode, a Celgard 2500 membrane separator, and 1 M LiPF_6_ dissolved in a mixed solvent of ethylene carbonate (EC), diethyl carbonate (DEC), and dimethyl carbonate (DMC) (1:1:1 by volume) serving as the electrolyte. Half-cell cycling and rate performances were assessed using a NEWARE battery test system (MHW-25-S-16CH, Shenzhen, China) over a 3.0-4.3 V potential range. Cyclic voltammetry (CV) tests were performed on a CHI760E electrochemical workstation.

**Characterization Methods**

The concentrations of various metal ions in the solutions were determined by inductively coupled plasma optical emission spectroscopy (ICP-OES, Optima 8300DV, PerkinElmer, USA). The morphologies of the cathode materials were measured by scanning electron microscopy (GeminiSEM 300, Zeiss). Fourier transform infrared spectroscopy (FTIR) was performed using a Nicolet iS50 FT-IR (Thermo-Fisher Scientific, USA). The crystal structure of the cathode materials was characterized by X-ray diffraction (XRD, D8 Bruker) with Cu Ka radiation (k = 1.5406 Å). In addition, in situ temperature-dependent XRD measurements were performed using a Rigaku smartlab 9 kW, with a heating rate of 5 °C min^-1^, to monitor the annealing process from room temperature to 850 °C. X-ray photoelectron spectroscopy (XPS) with an Al Kα source was applied to collect surface information through an XPS Escalab Xi+ spectrometer.

**COMSOL simulation**

In this study, the Transport of Diluted Species (tds) module in COMSOL Multiphysics was employed to perform the calculations, which adhere to Fick's law of diffusion. The particle size was set to 30 μm, and the thickness of the external damaged layer was defined as 3 μm. A free triangular mesh was adopted, consisting of 31,445 elements. The minimum element quality was 0.01321, with an average value of 0.8056.

$$\frac{\partial c}{\partial t}+\nabla J+u\nabla c=R$$

$$J=-D_{e}\nabla c$$

*C* represents the concentration of the mass-transfer fluid, *J* denotes the mass-transfer flux, *R* is the volumetric reaction source (with no reaction occurring in this model, thus *R* = 0), *D_e_* is the diffusion coefficient of the species, and *u* is the fluid velocity field. It is assumed that lithium ions react immediately upon reaching the damaged region; therefore, the distribution of lithium ions can be regarded as the spatial distribution of the repair degree. The damaged region is assigned a value of 0, while the undamaged region is assigned a value of 1. The contact interface is defined as the entry channel for lithium ions. On the non-pre-lithiated surface, the lithium-ion influx sites are sparse and dispersed, whereas on the pre-lithiated surface, lithium ions flow in uniformly. As the ions continuously inflow, the surface-supplied species are gradually consumed.

**
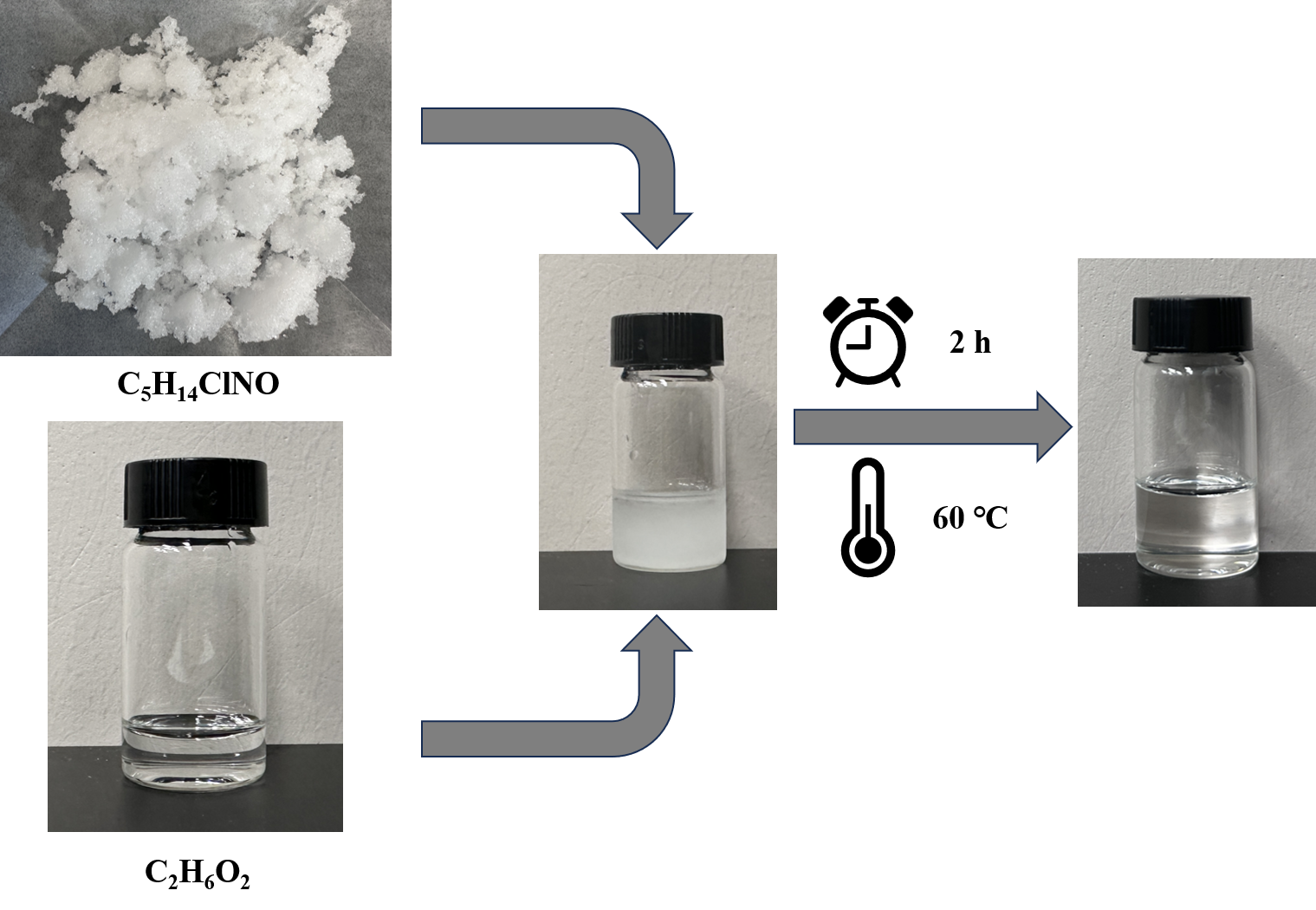
**

**Figure S1. Schematic illustration of the DES synthesis process.**

**
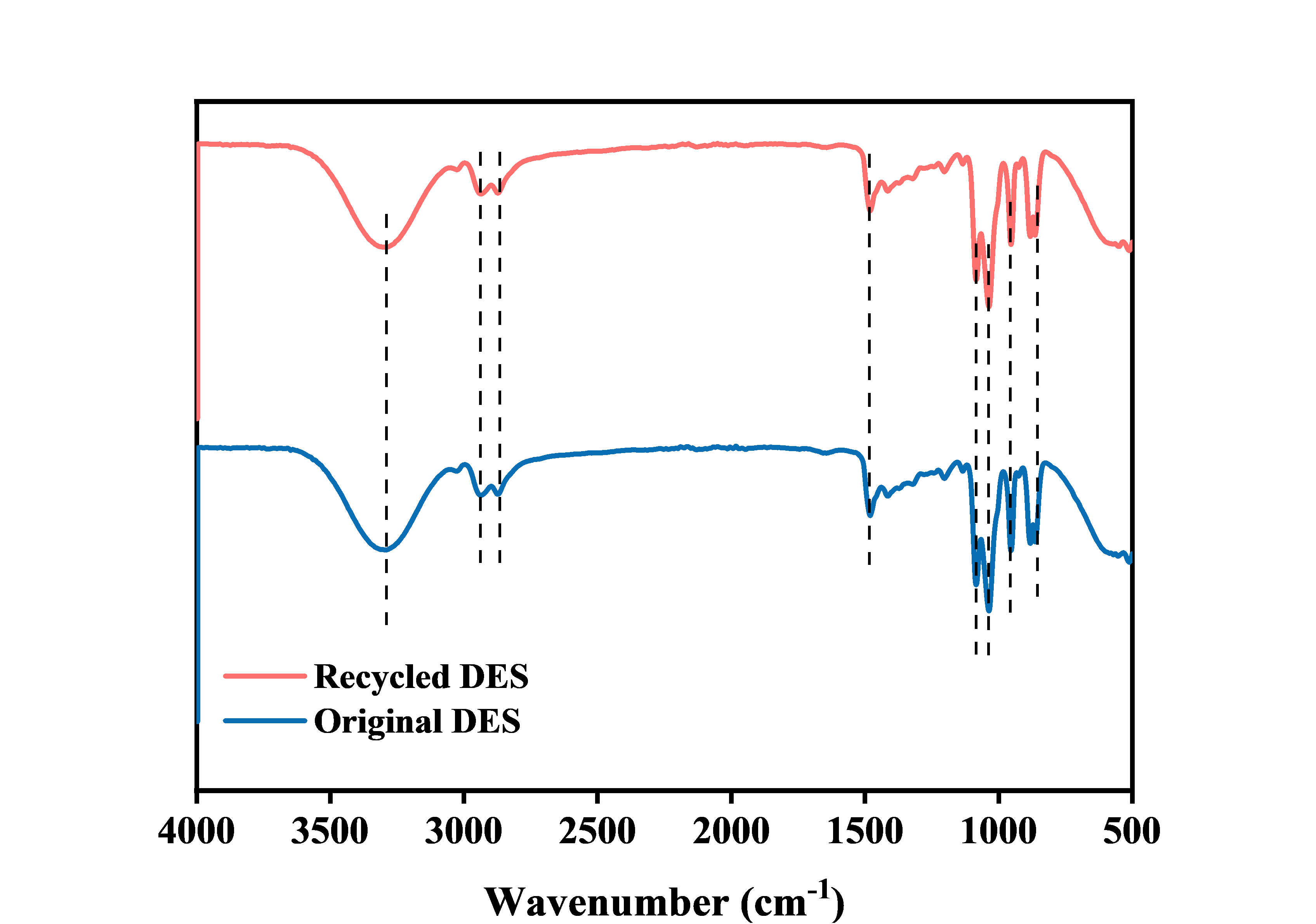
**

**Figure S2.** FTIR spectra of original and recycled DES.

**
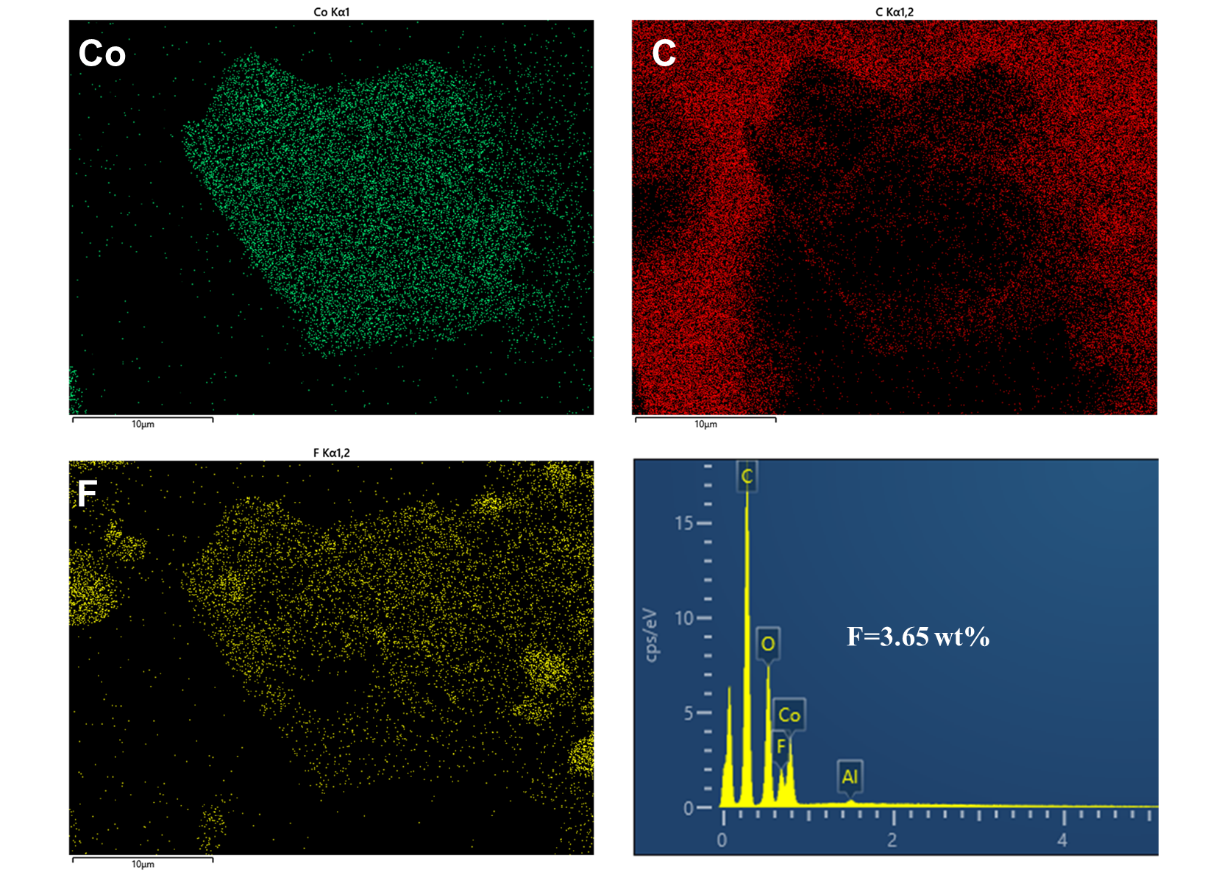
**

**Figure S3.** EDS spectra of Co, C, and F in SLCO.

**
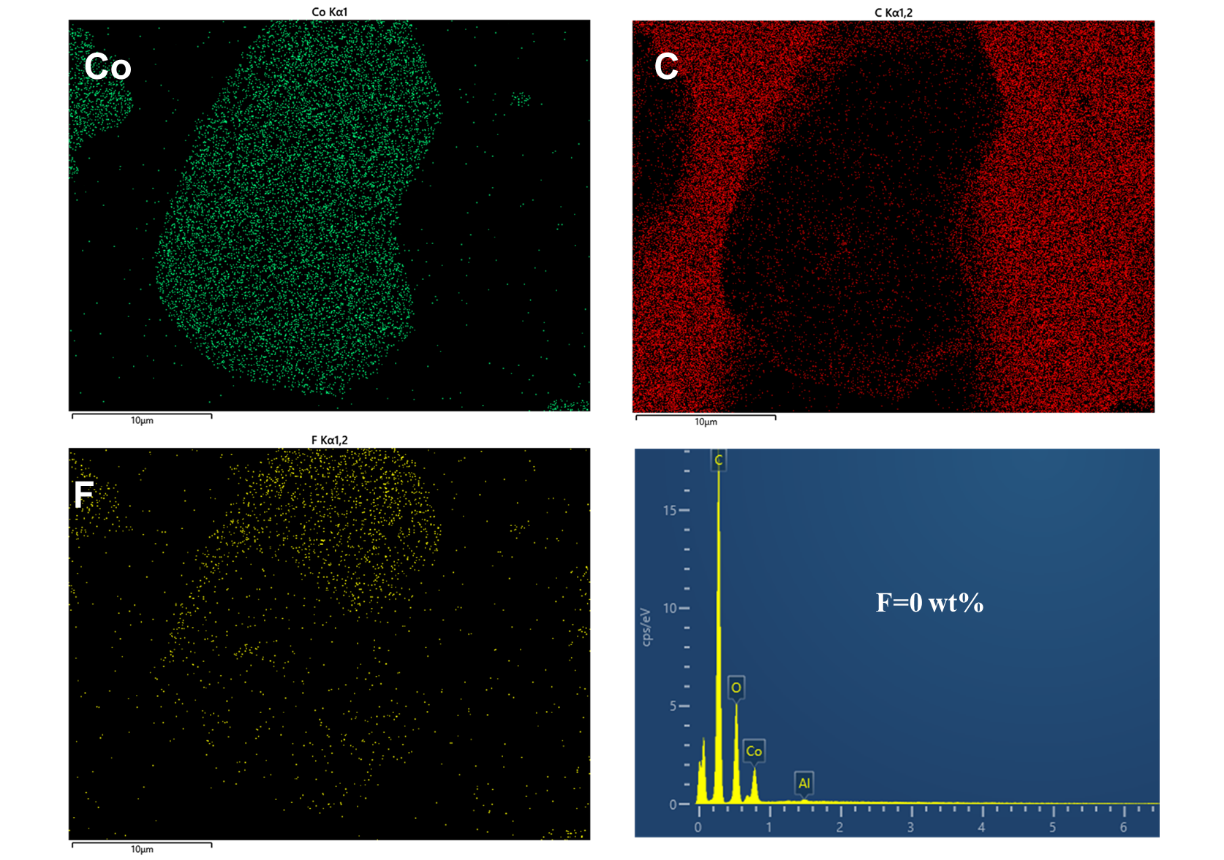
**

**Figure S4.** EDS spectra of Co, C, and F in DES-treated SLCO.

**
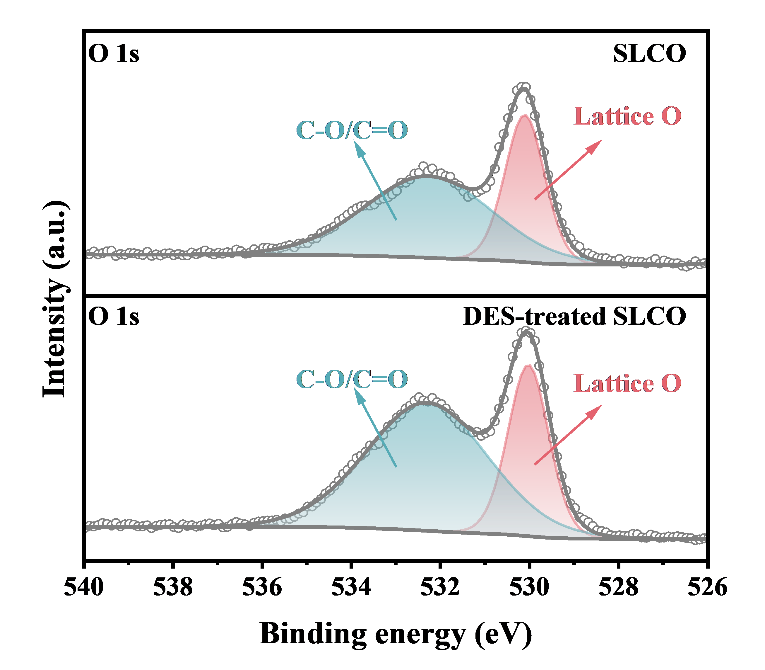
**

**Figure S5.** The XPS patterns of SLCO and DES-treated SLCO: O 1s.


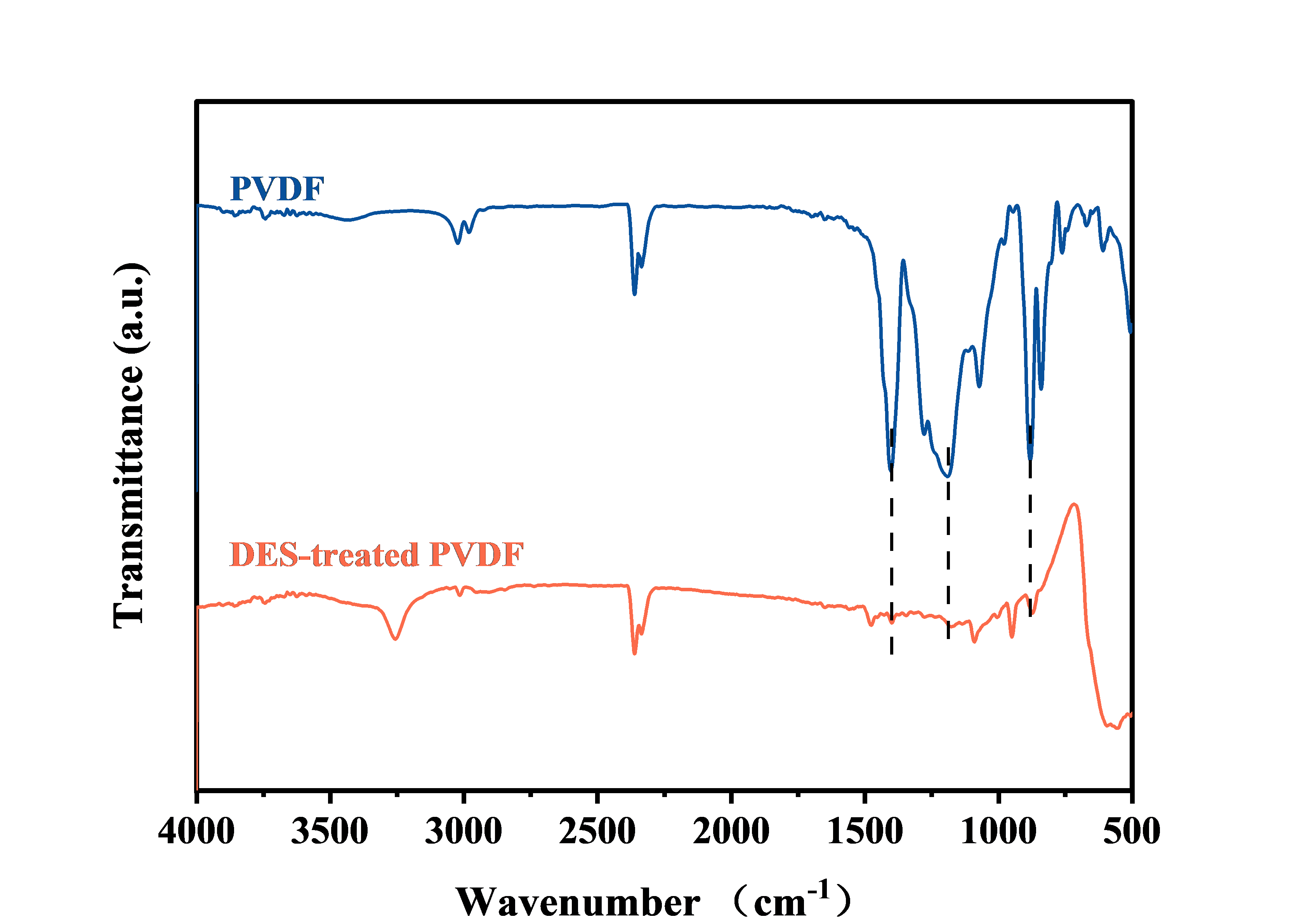


**Figure S6.** FT-IR spectra of PVDF and DES-treated PVDF.

**
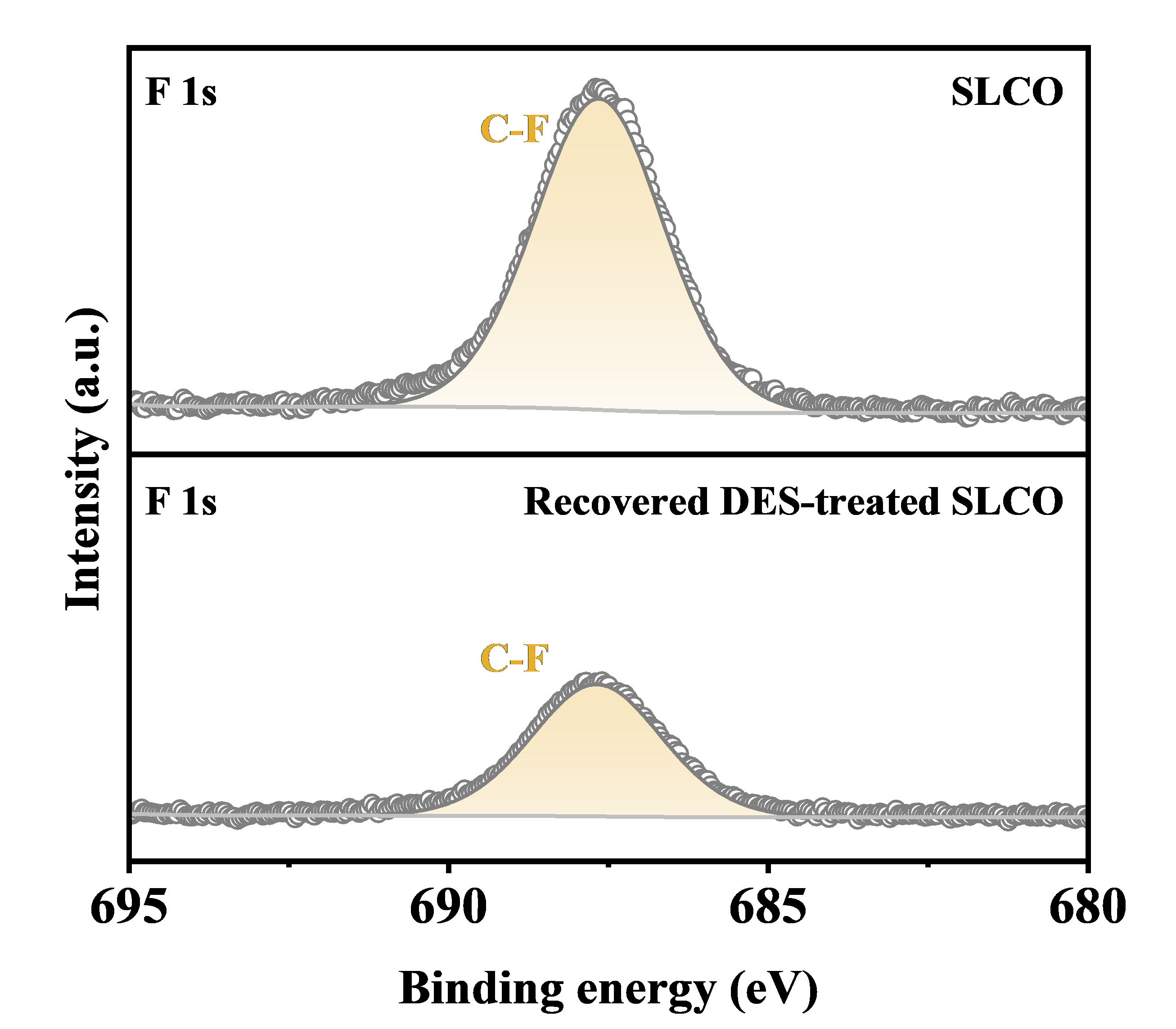
**

**Figure S7.** The XPS patterns of SLCO and Recovered DES-treated SLCO: F1s.

**
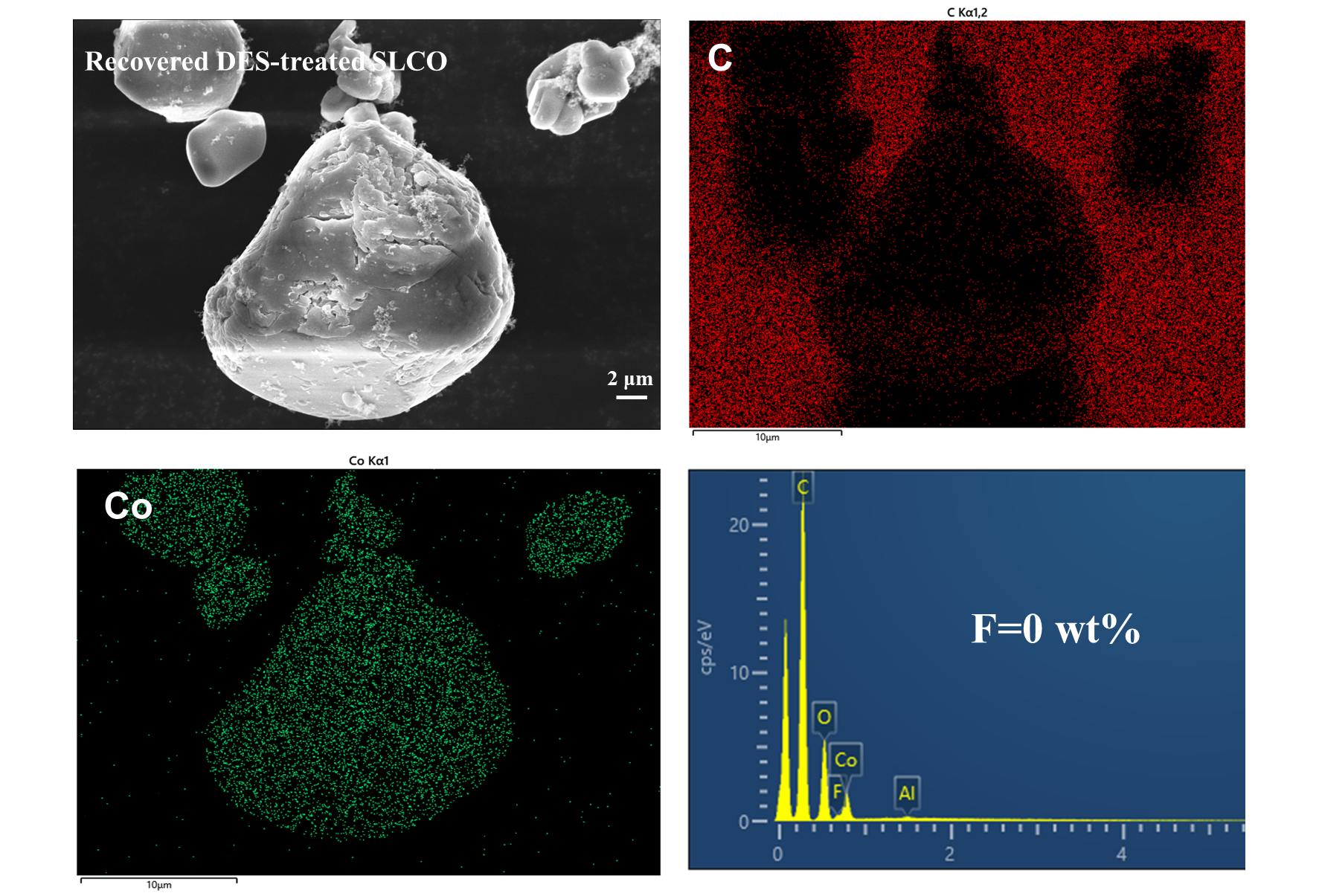
**

**Figure S8.** SEM-EDS analysis of SLCO after treatment with the recovered DES.

**
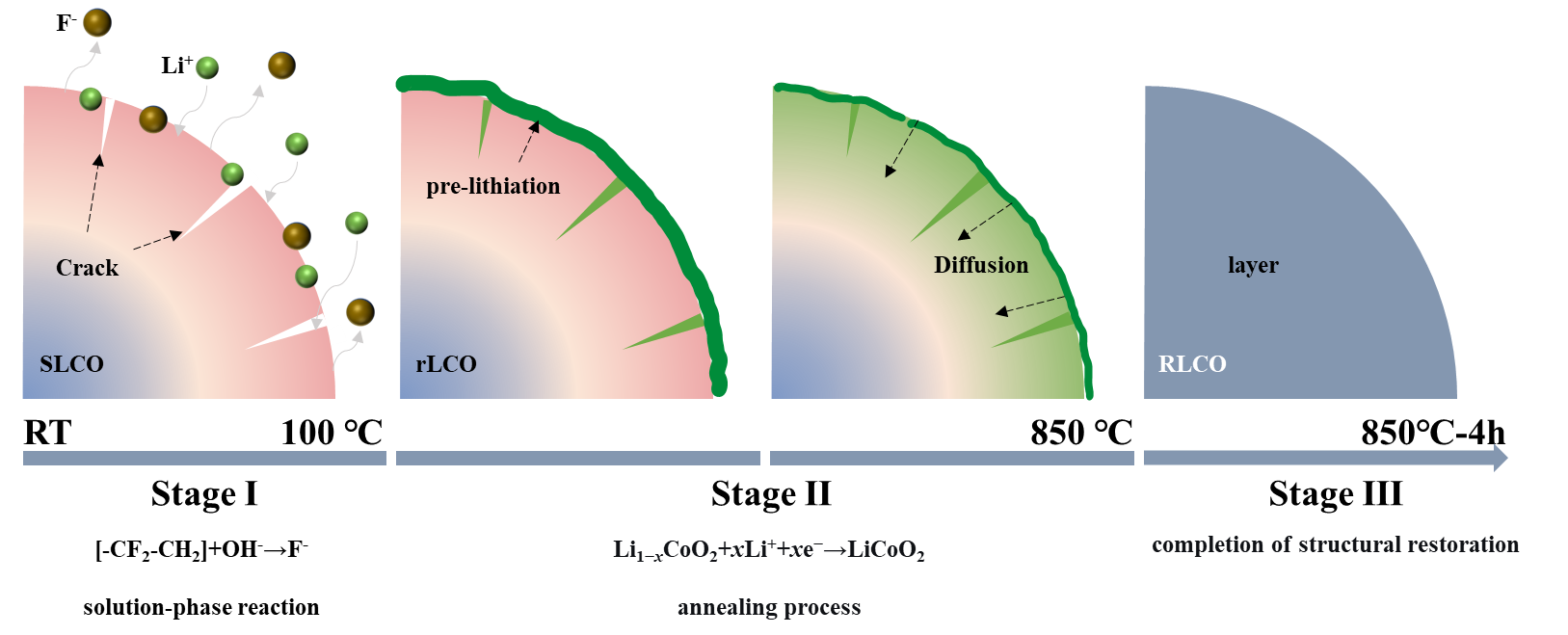
**

**Figure S9.** Schematic of the regeneration process.


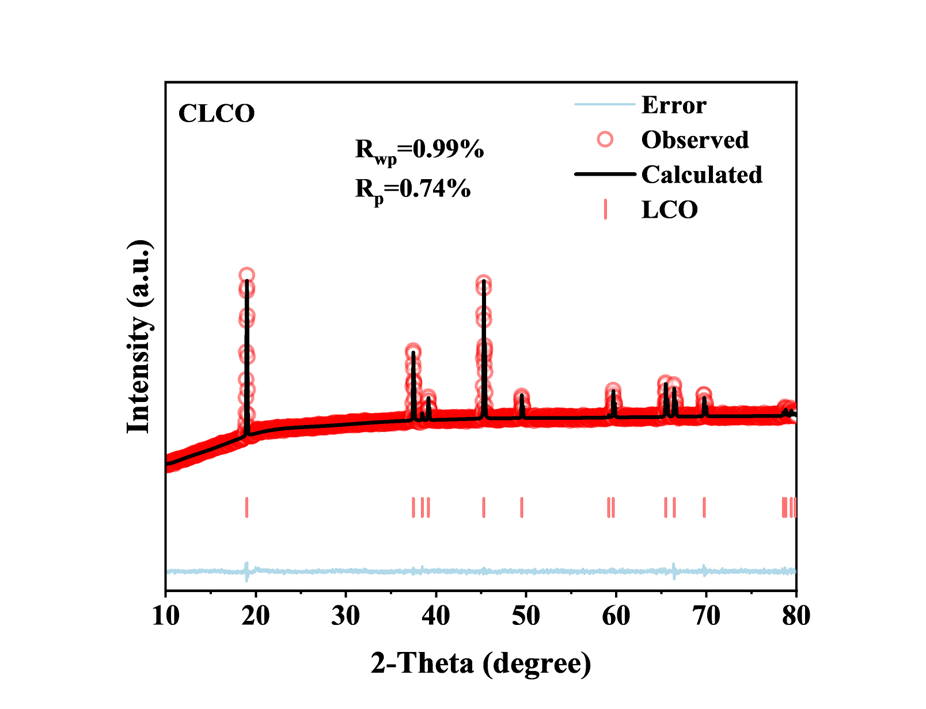


**Figure S10.** Rietveld refinement of the XRD pattern of CLCO.

**
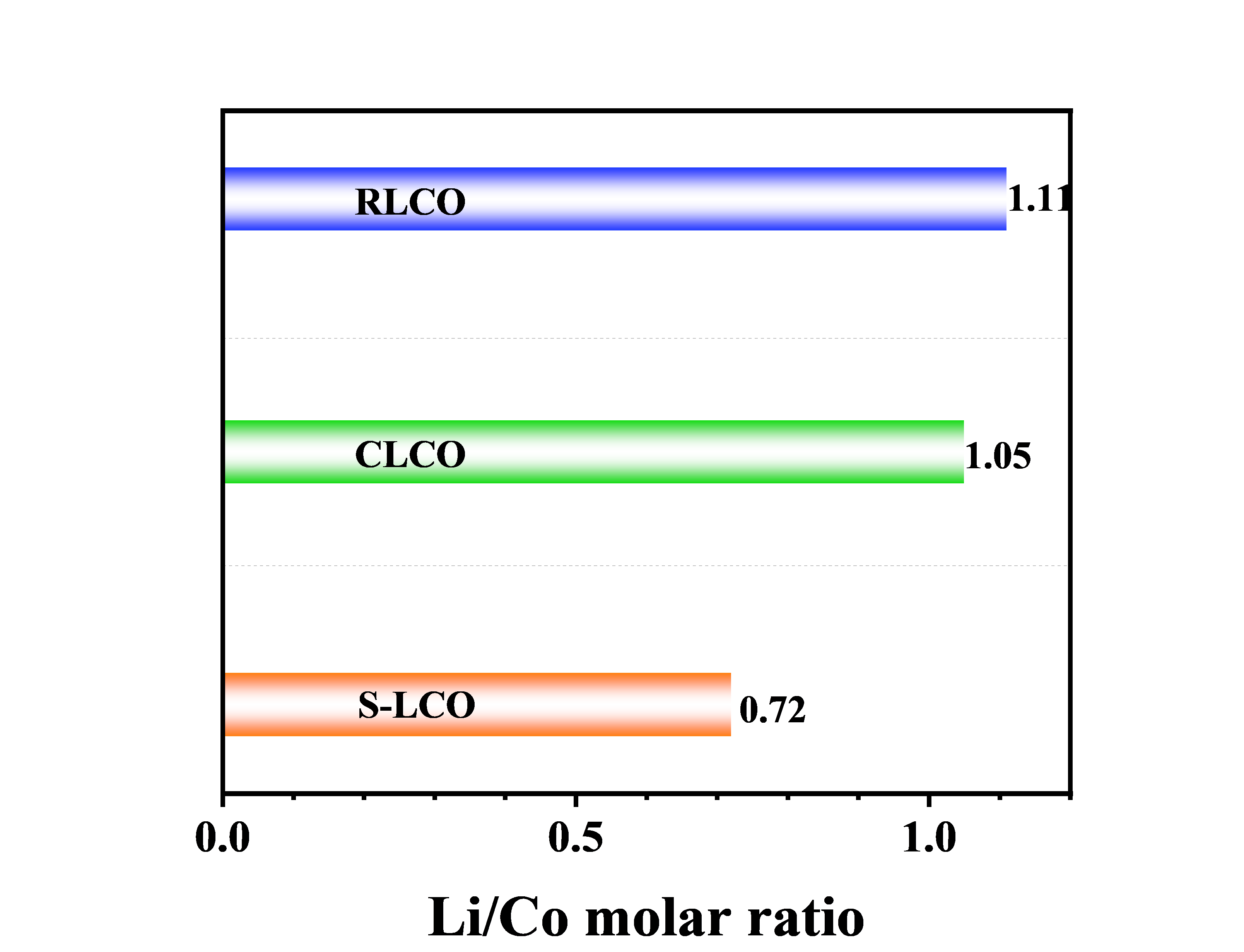
**

**Figure S11.** ICP of SLCO, CLCO, and RLCO.

**
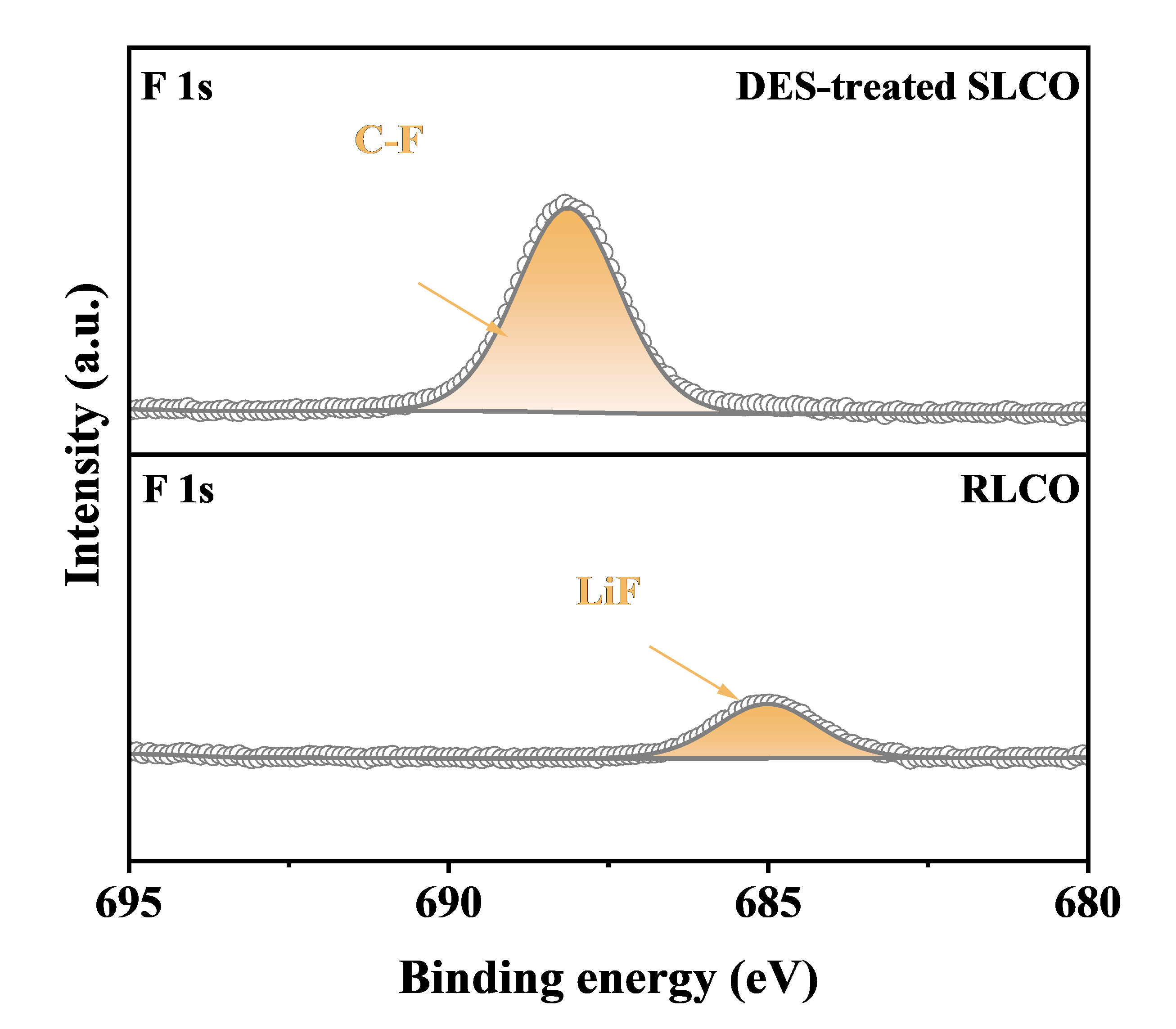
**

**Figure S12.** The XPS patterns of DES-treated SLCO and RLCO: F 1s.

**
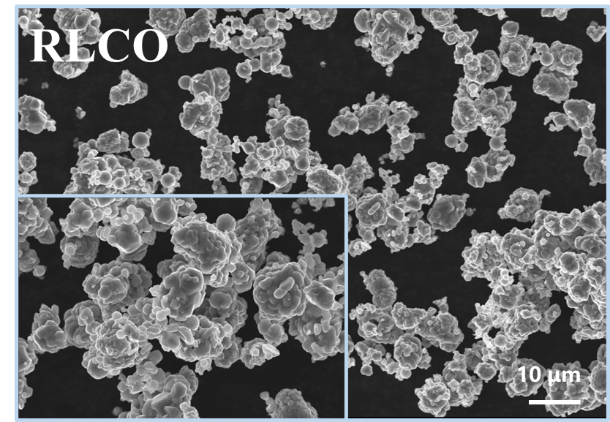
**

**Figure S13.** SEM images of RLCO.

**
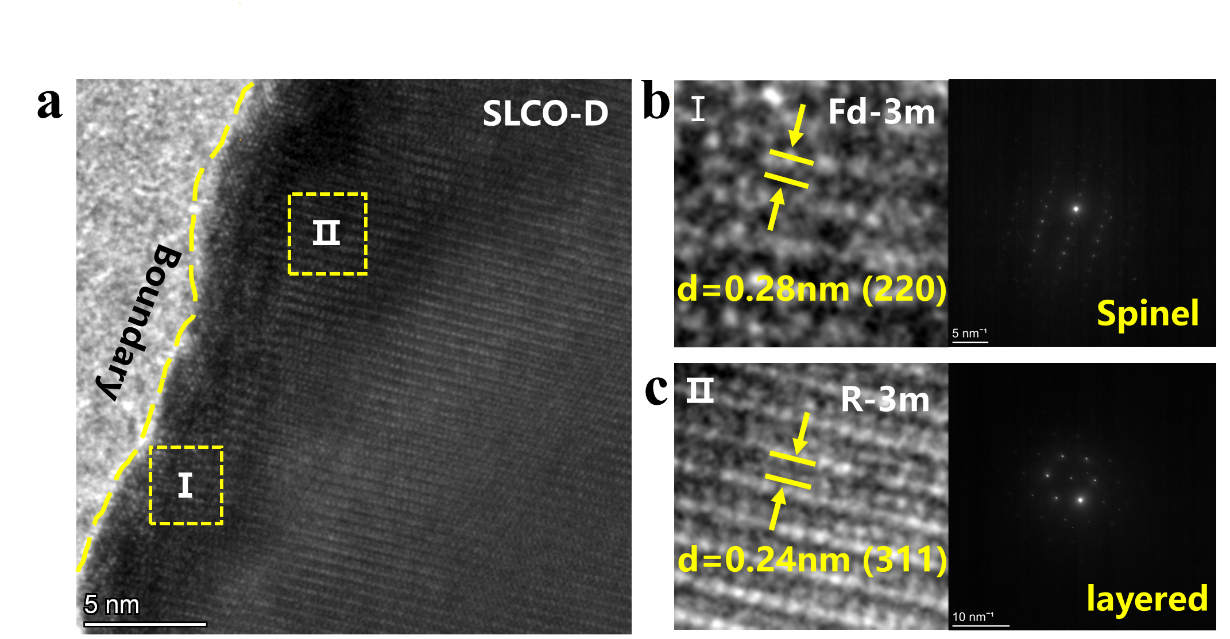
**

**Figure S14.** HRTEM and FFT images of SLCO-D.

**
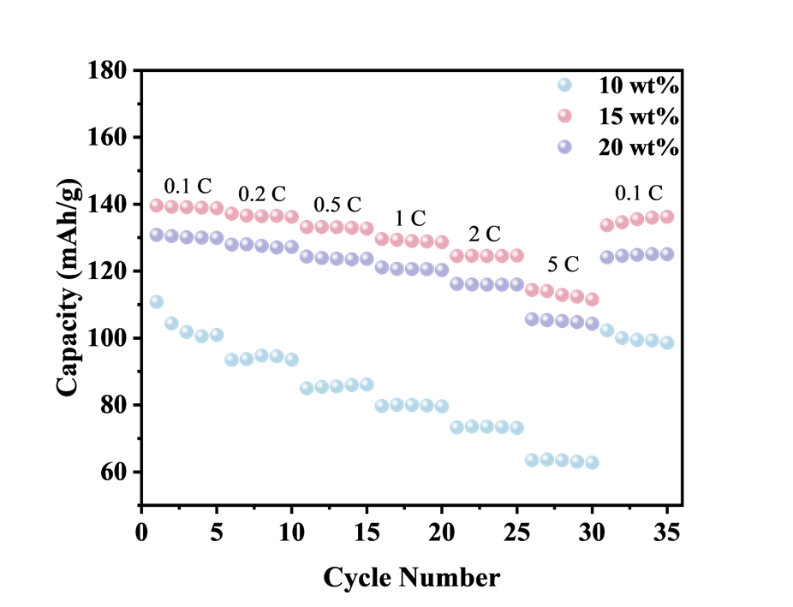
**

**Figure S15.** Rate performance at different lithium replenishment amounts.

**
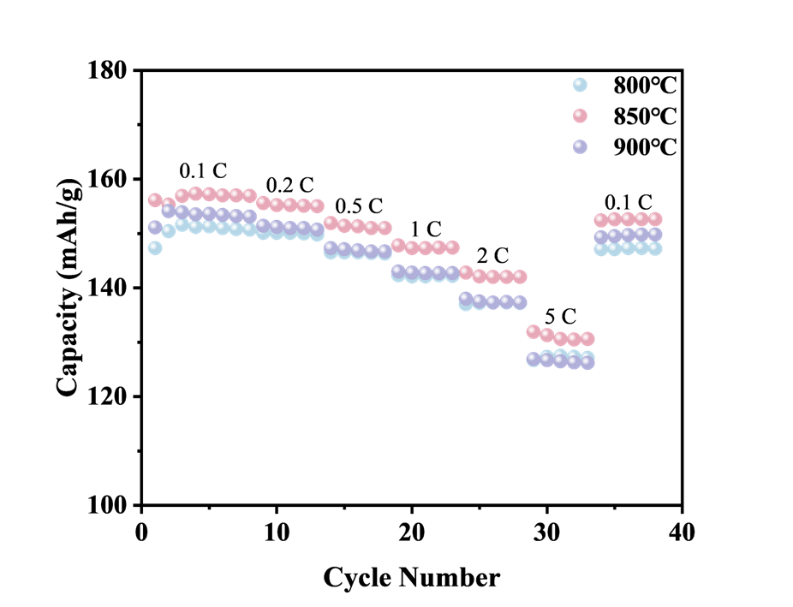
**

**Figure S16.** Rate performance at different annealing temperatures.


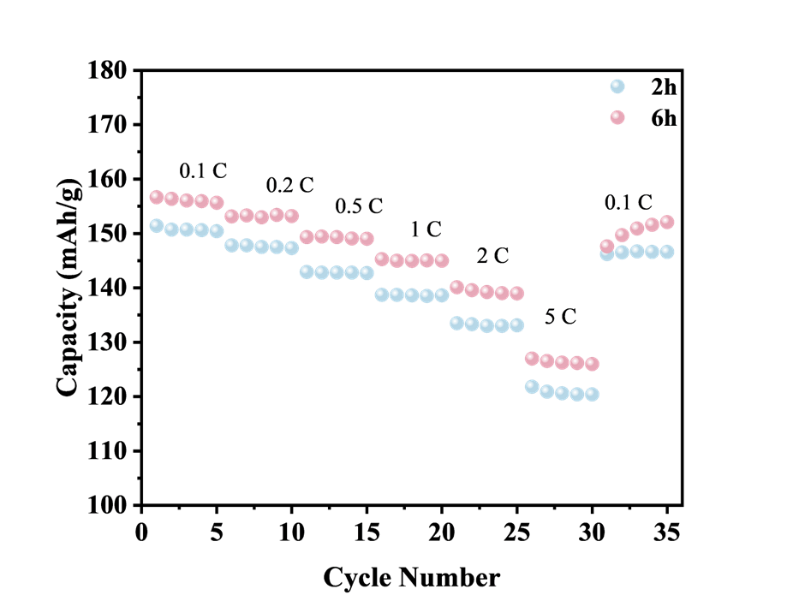


**Figure S17.** Rate performance as a function of reaction time.

**
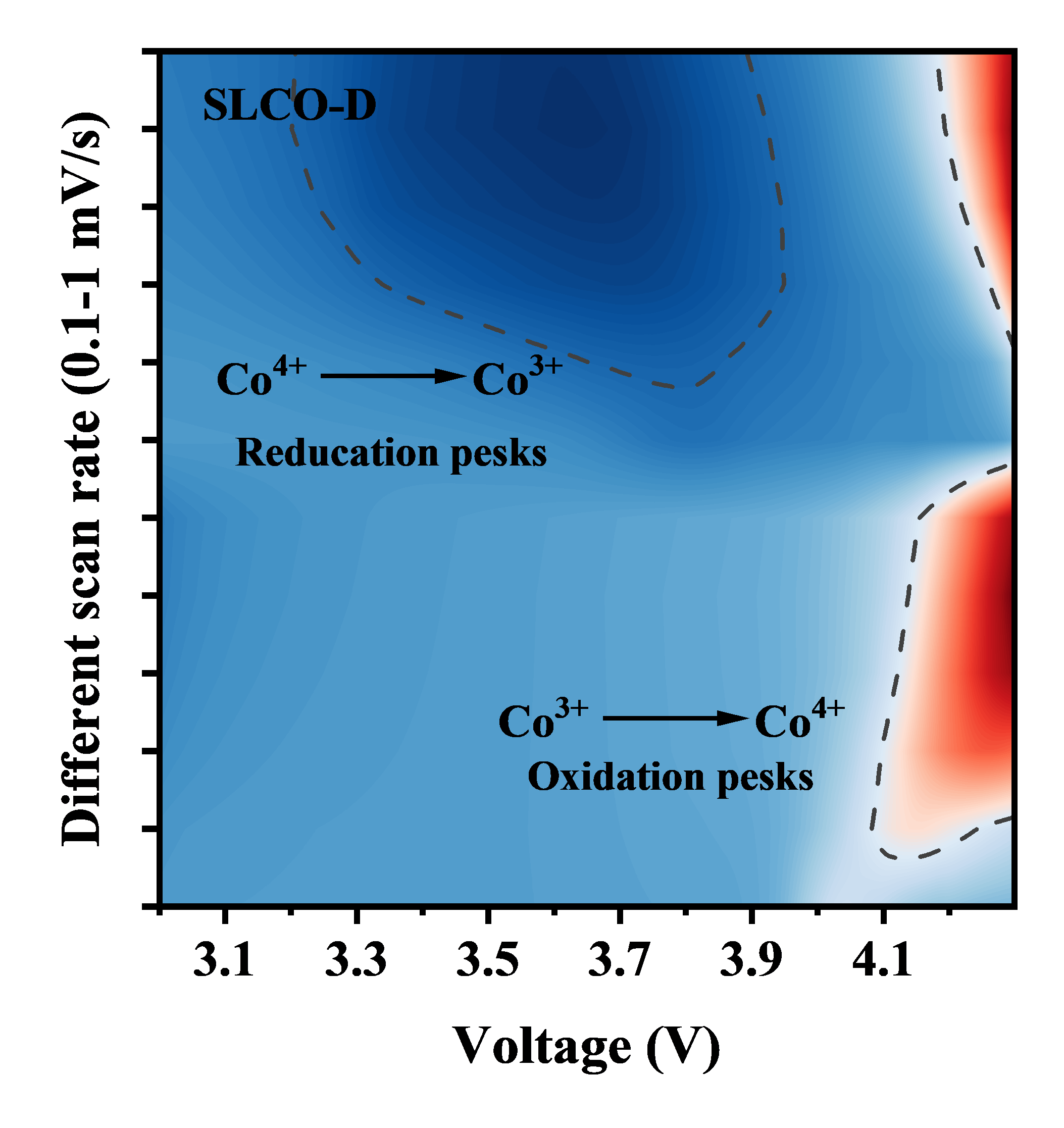
**

**Figure S18.** CV curves of SLCO-D.

**
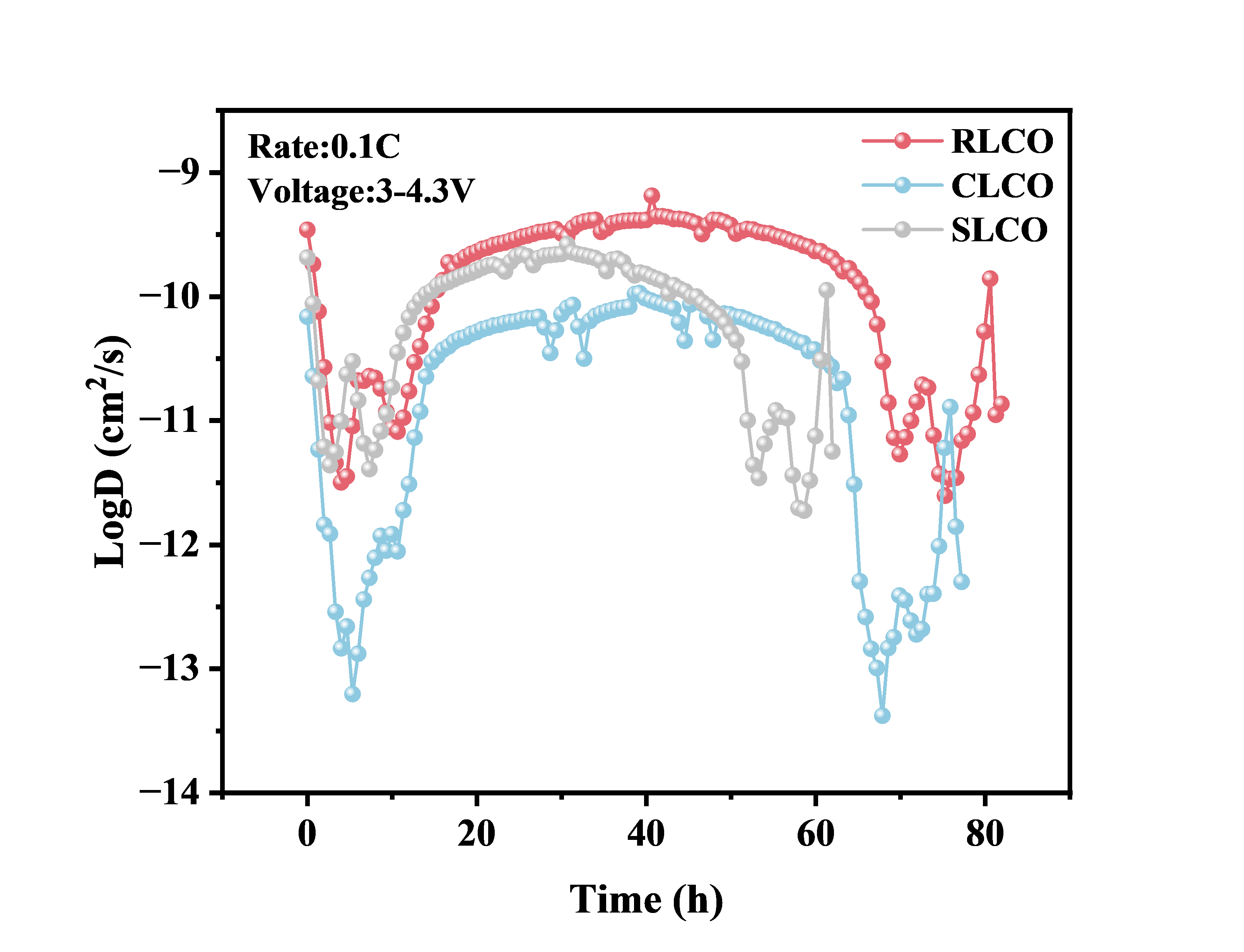
**

**Figure S19.** GITT measurement of SLCO, RLCO, and CLCO.

**
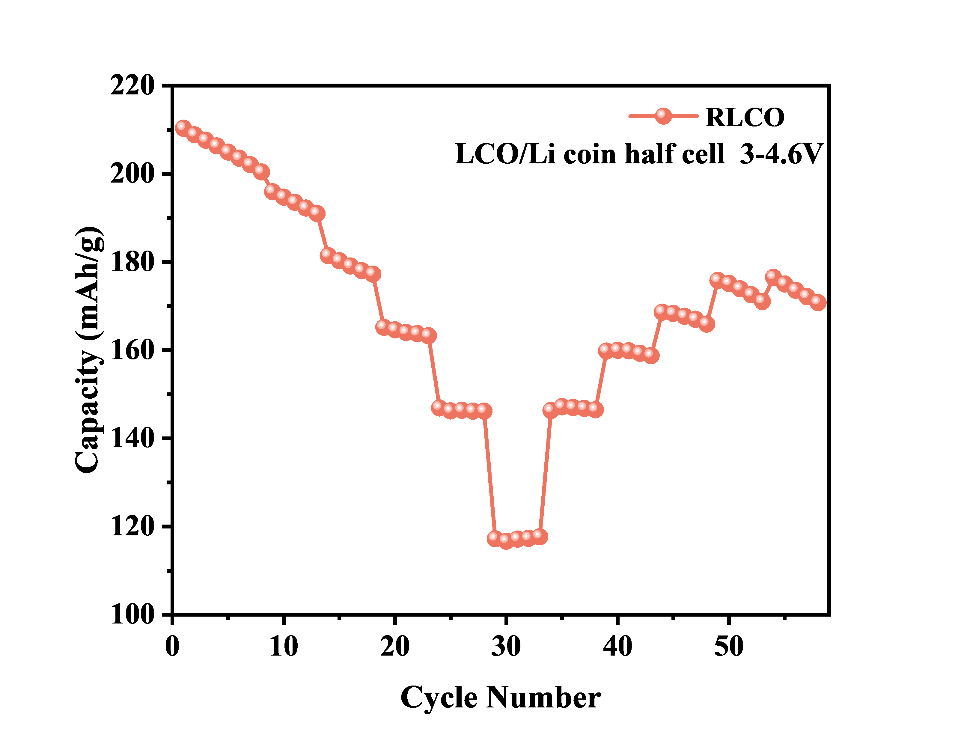
**

**Figure S20** High-Voltage Rate Capability of the Regenerated RLCO.

**
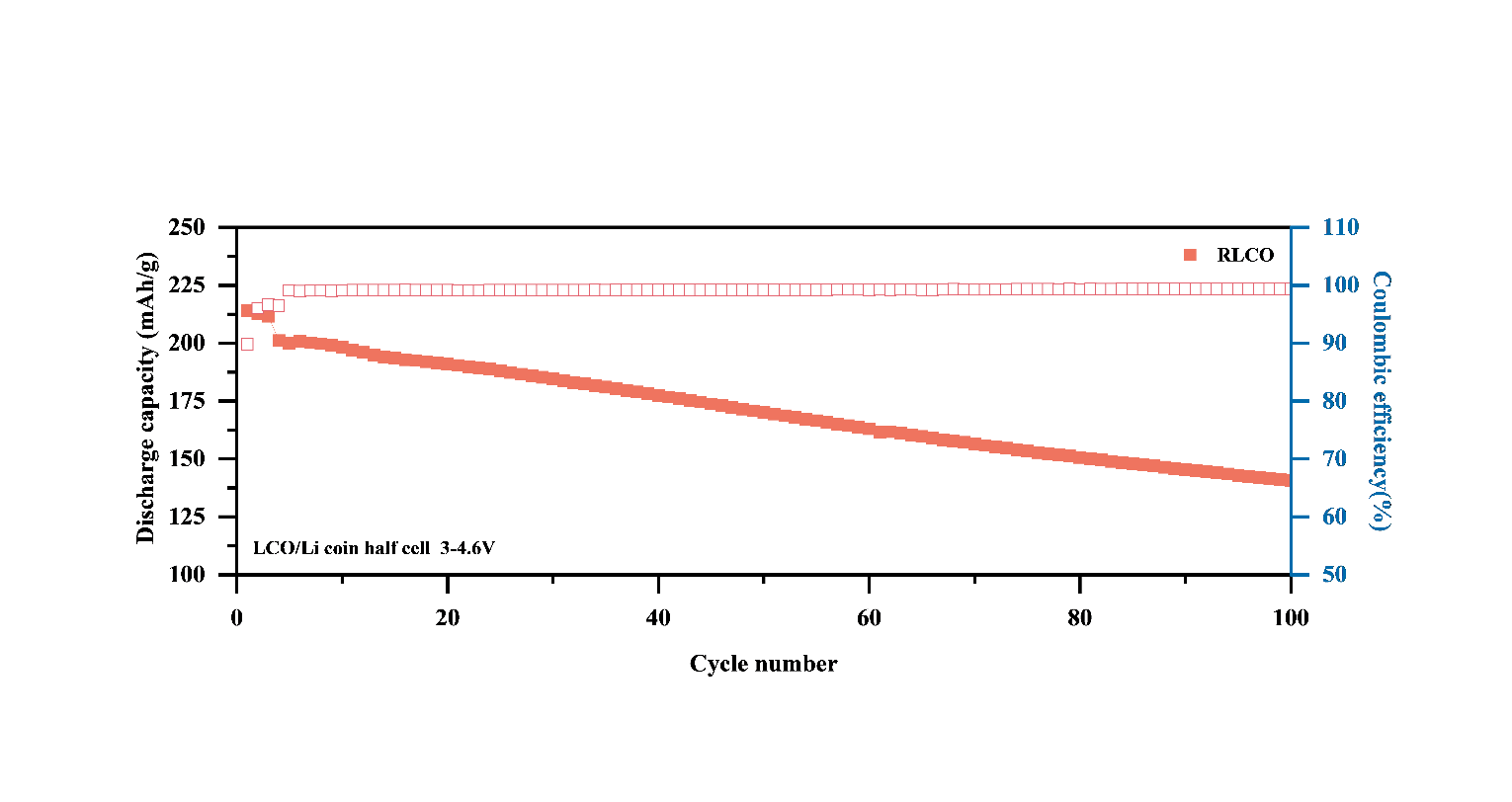
**

**Figure S21.** Cycling Performance of RLCO at High-Voltage.

**Table S1**. Rietveld refinement results of the XRD pattern of SLCO.

| Sample | a (Å) | b (Å) | c (Å) | Li/Co antisite |
| --- | --- | --- | --- | --- |
| S-LCO | 2.81010 | 2.81010 | 14.05122 | 4.86% |
| Space group: R-3m and Fd-3m  Phase composition: LCO 99.392%, Co_3_O_4_ 0.608%  Refinement results: R_wp_=1.01%, χ^2^=1.24% | | | | |

**Table S2**. Rietveld refinement results of the XRD pattern of RLCO.

| Sample | a (Å) | b (Å) | c (Å) | Li/Co antisite |
| --- | --- | --- | --- | --- |
| R-LCO | 2.81390 | 2.81390 | 14.05656 | 1.87% |
| Space group: R-3m  Refinement results: R_wp_=0.93%, χ^2^=1.47% | | | | |

**Table S3**. Rietveld refinement results of the XRD pattern of CLCO.

| Sample | a (Å) | b (Å) | c (Å) | Li/Co antisite |
| --- | --- | --- | --- | --- |
| C-LCO | 2.81399 | 2.81515 | 14.05666 | 1.75% |
| Space group: R-3m  Refinement results: R_wp_=1.00%, χ^2^=1.28% | | | | |

**Table S4**. Comparison of the cycling performance of RLCO with regenerated cathode materials from the literature.

| Cathode  materials | Voltage/V | Rate | Capacity  retention  after 50  cycles/% | Capacity  retention  after 100  cycles/% | Capacity  retention  after 200  cycles/% | Reference |
| --- | --- | --- | --- | --- | --- | --- |
| RLCO | 3-4.3 | 0.5C | 97.85 | 96.18 | 91.02 | This work |
| D-LCO 70% re-Li | 3-4.5 | 0.5C | 83% | / | / | [1] |
| NR-HSOH | 2.7-4.2 | 0.2C | 94% | / | / | [2] |
| NLCO0.01 | 3-4.3 | 1C |  | 95% |  | [3] |
| D-LCO-R-H | 2-4.2 | 0.5C |  | 90.0% | / | [4] |
| Regenerated LiCoO_2_ | 2.75-4.25 | 0.2C |  | 93.1% | / | [5] |
| Recycled LiCoO_2_ | 3-4.2 | 0.1C |  | 93% | / | [6] |
| R-LCO-Li-1440 | 3-4.3 | 0.2C |  | 86.7% | / | [7] |
| r-LCO-MA | 3-4.6 | 0.2C |  |  | 84% | [8] |
| Regenerated LiCoO_2_ | 2.75-4.25 | 0.2C |  |  | 92.5% | [9] |

**Table S5**. Preprocessing throughput information.

| **Preprocessing throughput** | | | |
| --- | --- | --- | --- |
| Chemistry | Type | tonne/yr | Geographic location |
| SLCO | Black mass | 10,000 | China |

**Table S6**. The usage and cost of consumed chemicals.

|  | Material | Usage (kg) | Unit price ($ per kg) |
| --- | --- | --- | --- |
| **Pyro-** | Limestone | 0.3 | 0.13 |
|  | Sand | 0.15 | 0.05 |
|  | Hydrochloric acid | 0.21 | 0.57 |
|  | Hydrogen peroxide | 0.06 | 1.46 |
| **Hydro-** | Sulfuric acid | 1.08 | 0.08 |
|  | Sodium hydroxide | 0.56 | 0.45 |
|  | Hydrochloric acid | 0.01 | 0.57 |
|  | Hydrogen peroxide | 0.37 | 1.46 |
|  | Ammonium hydroxide | 0.031 | 0.53 |
|  | Soda ash | 0.02 | 0.14 |
| **Li^+^-DES** | Lithium hydroxide | 0.05 | 19 |
|  | Choline chloride | 5.29 | 1.3 |
|  | Ethylene glycol | 4.71 | 0.1 |

Note: The unite price is obtained from Everbatt 2023 database.

**Table S7**. The price of materials used by Hydro- and direct recycling.

|  | Products | Quantity (kg) | Unit price ($ per kg) |
| --- | --- | --- | --- |
| **Pyro-** | Copper metal | 0.01 | 7.11 |
|  | Co^2+^ in product | 0.4 | 50.35 |
| **Hydro-** | Lithium Carbonate (crude) | 0.237 | 8.57 |
|  | Co^2+^ in product | 0.414 | 50.35 |
|  | Graphite | 0.237 | 0.2 |
| **Li^+^-DES** | LCO | 0.51 | 50 |
|  | Graphite | 0.24 | 0.2 |

Note: The value of recycled materials was obtained from the Everbatt 2023 database.

**Table S8**. Recycling cost ($ per kg feedstock) of different battery recycling technologies.

|  | **Pyro-** | **Hydro-** | **Li^+^-DES** |
| --- | --- | --- | --- |
| Materials | 15.61 | 16.28 | 17.15 |
| Utilities | 0.12 | 0.03 | 0.07 |
| Other variable costs | 0.31 | 0.32 | 0.34 |
| Labor | 0.03 | 0.03 | 0.05 |
| Maintenance | 0.24 | 0.12 | 0.09 |
| Plant overhead | 0.41 | 0.44 | 0.48 |
| Other fixed costs | 0.57 | 0.43 | 0.4 |
| Annualized capital cost | 1.49 | 0.75 | 0.54 |

**Table S9**. Recycling revenue ($ per kg feedstock) of different battery recycling technologies.

|  | **Pyro-** | **Hydro-** | **Li^+^-DES** |
| --- | --- | --- | --- |
| Copper metal | 0.08 |  |  |
| Co^2+^ in product | 20.22 | 20.86 |  |
| Lithium Carbonate (crude) |  | 2.03 |  |
| Graphite |  | 0.05 | 0.05 |
| LCO |  |  | 25.5 |
|  | 20.31 | 22.94 | 25.55 |

**Table S10**. Recycling profit ($ per kg feedstock) of different battery recycling technologies.

|  | **Pyro-** | **Hydro-** | **Li^+^-DES** |
| --- | --- | --- | --- |
| Cost | 18.79 | 18.41 | 19.11 |
| Revenue | 20.31 | 22.94 | 25.55 |
| Profit | 1.52 | 4.53 | 6.44 |

**Table S11**. GHG emission (g per kg feedstock) and total energy consumption (MJ per kg feedstock) of different battery recycling technologies.

|  | **Pyro-** | **Hydro-** | **Li^+^-DES** |
| --- | --- | --- | --- |
| GHGs | 2616 | 1963 | 1768 |
| Total energy | 19.980 | 30.355 | 21.572 |

# **Reference**

[1] K. Lahtinen, E.-L. Rautama, H. Jiang, S. Räsänen, T. Kallio, *ChemSusChem* **2021**, *14* (11), 2434, <https://doi.org/https://doi.org/10.1002/cssc.202100629>.

[2] X. Mu, K. Huang, G. Zhu, Y. Li, C. Liu, X. Hui, M. Sui, P. Yan, *Nano Energy* **2023**, *112*, 108465, <https://doi.org/https://doi.org/10.1016/j.nanoen.2023.108465>.

[3] J. Wu, J. Lin, E. Fan, R. Chen, F. Wu, L. Li, *ACS Applied Energy Materials* **2021**, *4* (3), 2607, <https://doi.org/10.1021/acsaem.0c03192>.

[4] J. Wang, Q. Zhang, J. Sheng, Z. Liang, J. Ma, Y. Chen, G. Zhou, H.-M. Cheng, *National Science Review* **2022**, *9* (8), nwac097, <https://doi.org/10.1093/nsr/nwac097>.

[5] H. Yang, B. Deng, X. Jing, W. Li, D. Wang, *Waste Management* **2021**, *129*, 85, <https://doi.org/https://doi.org/10.1016/j.wasman.2021.04.052>.

[6] T. Yang, Y. Lu, L. Li, D. Ge, H. Yang, W. Leng, H. Zhou, X. Han, N. Schmidt, M. Ellis, Z. Li, **2020**, *4* (1), 1900088, <https://doi.org/https://doi.org/10.1002/adsu.201900088>.

[7] Y.-C. Yin, C. Li, X. Hu, D. Zuo, L. Yang, L. Zhou, J. Yang, J. Wan, *ACS Energy Letters* **2023**, *8* (7), 3005, <https://doi.org/10.1021/acsenergylett.3c00635>.

[8] Y. Cheng, J. Chen, W. Chen, Q. Liu, O. E. Onah, Z. Wang, G. Wu, T. Xie, L. Eddy, B. I. Yakobson, J. Li, Y. Zhao, J. M. Tour, *Energy & Environmental Science* **2025**, *18* (12), 6085, <https://doi.org/10.1039/D5EE00962F>.

[9] J. Yang, W. Wang, H. Yang, D. Wang, *Green Chemistry* **2020**, *22* (19), 6489, <https://doi.org/10.1039/D0GC02662J>.
